# CDCA3 is a prognostic biomarker for cutaneous melanoma and is connected with immune infiltration

**DOI:** 10.3389/fonc.2022.1055308

**Published:** 2023-01-11

**Authors:** Tianhao Li, Liquan Wang, Nanze Yu, Ang Zeng, Jiuzuo Huang, Xiao Long

**Affiliations:** Department of Plastic and Cosmetic Surgery, Peking Union Medical College Hospital, Chinese Academy of Medical Sciences & Peking Union Medical College, Beijing, China

**Keywords:** cell cycle progression derived gene, melanoma, cell division cycle-associated gene 3, LASSO, TCGA, GEO

## Abstract

**Introduction:**

Dysregulation of cell cycle progression (CCP) is a trait that distinguishes cancer from other diseases. In several cancer types, CCP-related genes serve as the primary risk factor for prognosis, but their role in cutaneous melanoma remains unclear.

**Methods:**

Data from cutaneous melanoma patients were acquired from The Cancer Genome Atlas (TCGA) and Gene Expression Omnibus (GEO). Using a Wilcoxon test, the level of CCP-related gene expression in cutaneous melanoma patient tissues was compared to that in normal skin tissues. Logistic analysis was then utilized to calculate the connection between the CCP-related genes and clinicopathological variables. The important functions of the CCP-related genes were further investigated using Gene Ontology (GO), Kyoto Encyclopedia of Genes and Genomes (KEGG) pathway analysis, and single-sample Gene Set Enrichment Analysis (ssGSEA). Univariate and multivariate Cox analyses and Kaplan–Meier analysis were used to estimate the association between CCP-related genes and prognosis. In addition, using Cox multivariate analysis, a nomogram was constructed to forecast the influence of CCP-related genes on survival rates.

**Results:**

High expression of CCP-related genes was associated with TNM stage, age, pathological grade, and Breslow depth (P < 0.05). Multivariate analysis demonstrated that CCP-related genes were an independent factor in overall survival and disease-specific survival. High levels of gene expression originating from CCP were shown by GSEA to trigger DNA replication, the G1-S specific transcription factor, the mitotic spindle checkpoint, and the cell cycle. There was a negative association between CCP-related genes and the abundance of innate immune cells. Finally, we revealed that knockdown of cell division cycle-associated gene 3 (CDCA3) significantly suppressed the proliferation and migration ability of cutaneous melanoma cells.

**Conclusion:**

According to this study, CCP-related genes could serve as potential biomarkers to assess the prognosis of cutaneous melanoma patients and are crucial immune response regulators.

## Introduction

Cutaneous melanoma is a lethal cutaneous cancer with a poor 5-year survival rate, the frequency of which has been increasing yearly (1). The management of advanced cutaneous melanoma has undergone a revolutionary change as a result of better knowledge of the mutational landscapes of cutaneous melanoma, the immunological response to cutaneous melanoma, and the related signaling pathways (2). However, drug resistance to signaling molecule inhibitors eventually develops and has a negative impact on the median progression-free survival of patients (3). In addition to the tumor staging system, there is an urgent need for biomarkers capable of identifying cutaneous melanoma, determining prognosis and monitoring the risk of metastases (4). Despite significant research and the identification of several clinical indicators and gene signatures to determine the prognosis of cutaneous melanoma (5–8), meta-analysis findings have shown that the prognostic ability of the gene expression profile is poor and varies by tumor stage (8), and the mechanisms underlying the effect of specific genes on prognosis in tumors remain elusive.

Cell cycle progression is a crucial biological process that is tightly regulated in healthy cells but almost always becomes aberrant or dysregulated in tumor cells (9, 10). There is evidence that CCP-related genes may be valuable biomarkers for predicting cancer recurrence and metastasis (11, 12). Cell cycle progression-related genes play a major role in controlling the progression of the cell cycle. Different human tumor processes frequently exhibit abnormalities in CCP-related gene expression, and their expression patterns have extensive prognostic value.

Cell division cycle-associated gene 3 (CDCA3) was first identified as a modulator of cell cycle progression for initiation of mitosis from G2 phase (13). The cell cycle is modulated by a family of genes associated with cell division. A number of studies have indicated that dysfunction of CDCA genes can influence the infiltration of immune cells in tumors as well as cell proliferation, leading to carcinogenesis (14). Studies have suggested increased CDCA3 expression in many cancer types (14–16). However, the effect of CDCA3 on prognosis in cutaneous melanoma remains unclear.

This study aimed to clarify the association of CCP- related genes with cutaneous melanoma based on comprehensive bioinformatics analysis and *in vitro* experiments. We applied RNA-seq data from TCGA and GEO datasets to conduct statistical and bioinformatics analyses, such as differentially expressed gene (DEG) analysis, KM survival analysis, Cox and logistic regression analysis, a nomogram, GO/KEGG analyses, and GSEA. Moreover, we knocked down the expression of CDCA3 *in vitro* to detect the impact on the proliferation and migration of cutaneous melanoma cells. Our findings contribute to uncovering the multifaceted roles of CCP-related genes and provide evidence for future gene-based therapy against cutaneous melanoma.

## Methods

### Data acquisition

RNAseq datasets of cutaneous melanoma from The Cancer Genome Atlas (https://portal.gdc.cancer.gov/), Gene Expression Omnibus (https://www.ncbi.nlm.nih.gov/geo), Genotype-Tissue Expression (https://www.gtexportal.org/home), and MSigDB database (http://software.broadinstitute.org/gsea/index.jsp) were used in this study. A total of 469 samples of cutaneous melanoma from TCGA, 812 samples of normal skin from GTEx, 528 CCP-related genes from MSigDB database and the GEO datasets GSE15605 (16 normal skin and 58 cutaneous melanoma) and GSE19234 (44 samples of cutaneous melanoma) were retrieved.

### Differences in gene expression

Gathering the TCGA, GTEx, GSE15605 and CCP- related gene data, the Limma program was used to identify differentially expressed genes (DEGs). |Fold change| >1.5 and false discovery rate (FDR)<0.05 were set as the cutoffs for the DEGs. Venn diagrams were used to display the three datasets, and the overlapping DEGs were chosen as potential genes.

### Enrichment analysis

We performed Gene Ontology (GO) enrichment analysis (BP: biological process; CC: cellular component; MF: molecular function) and Kyoto Encyclopedia of Genes and Genomes (KEGG) pathway analysis on the screened prognosis-associated genes using the R package cluster profiles. To identify functional categories and pathways, a P<0.05 cutoff value was employed. We used GSEA to analyze CDCA3-associated pathways and phenotypes by comparing the biofunction pathways between CDCA3-low and -high patients. A permutation test was used 1,000 times to assess relevant signaling pathways. Significantly related genes were defined by FDR<0.25 and adjusted P<0.01. R package ClusterProfiler was used to create graphic plots and conduct statistical analyses.

### Construction and validation of a risk signature associated with survival in cutaneous melanoma patients

The best gene combination for creating the risk signature was further identified using least absolute shrinkage and selection operator (LASSO) regression analysis. Genes that were substantially related to the overall survival (OS) of cutaneous melanoma patients were found using univariate Cox regression. The risk signature was constructed using regression coefficient-weighted pseudogene expression as follows: Risk score (RS) = (each variable gene’s expression× corresponding regression coefficient) + (expr2 × Coef2) + … + (exprn × Coefn). According to the median value of the RS, cutaneous melanoma patient subgroups were divided into high- and low-risk categories. Using the survminer program, the overall survival rates of the two subgroups were compared. By adjusting clinicopathological factors, Cox regression models were created to evaluate the gene signature’s prognostic independence. To assess the gene signature’s prediction ability using the survival ROC program, time-dependent ROC curve tests were carried out. The GEO dataset GSE19234 was used for external validation of the CCP- related gene signature.

### Establishment of a prognostic nomogram of clinical relevance

The rms package was used to construct a nomogram. The sum of the stage points, the total points, and the RS were used to evaluate 2- and 4-year overall survival. By using ROC, calibration, and conclusive curves, the predictive ability of this nomogram was confirmed. The clinical correlation analysis of TNM stage, age, race, pathological grade, and Breslow depth was obtained based on the risk score.

### Evaluation of immune-related cell infiltration

The relative infiltration levels of 24 different types of immune cells were quantified using expression data from published gene lists to more thoroughly study the tumor infiltration levels of immune cells. The applicable signatures consist of 509 genes and include a variety of innate and adaptive immune cell types (17). To evaluate the association between the infiltration levels of immune cells and CCP-related genes and the correlation of the different CCP-related gene expression groups with the infiltration of immune cells, Spearman correlation and Wilcoxon tests were used.

### Cell culture and transfection

Human skin melanoma cell lines (A375 and SK-MEL-2) were purchased from the American Type Culture Collection (Virginia, USA). Dulbecco’s modified Eagle’s medium (DMEM; HyClone) was used to cultivate melanoma cell lines at 37°C with 5% CO^2^. The DMEM was supplemented with fetal calf serum (10%, Thermo Fisher Scientific), penicillin (100 U/mL), and streptomycin (0.1 mg/mL). Cells in log phase were subsequently digested by trypsin and seeded into 6-well plates. Cell transfection experiments were conducted when cells reached 80% confluence according to the instructions for the Lipofectamine 3000 transfection kit. A375 cells were transfected with si-CDCA3 (5′-TGAAGACAGTGTCCTCATA-3′) to knock down CDCA3. Cells transfected with si-control were used as a negative control. The expression level of CDCA3 was measured 48 h after transfection.

### Western blotting

Total protein was isolated from cell lines and then separated *via* 10% sodium dodecyl sulfate−polyacrylamide gel electrophoresis (SDS−PAGE). Following transfer of the separated protein to a polyvinylidene fluoride membrane, the membrane was incubated for one hour with 5% nonfat dry milk to block nonspecific binding. Primary antibodies against CDCA3 (PTG: 15594-1-AP), C-Myc (PTG: 10828-1-AP), CyclinD1 (PTG: 60186-1-Ig), and Vinculin (PTG: 66305-1-Ig) were incubated with the membranes overnight at 4°C. The membranes were then incubated with secondary antibody for 1 hour after being washed with TBST three times (each time for 5 min). Finally, the membranes were visualized using ECL reagent (Thermo Fisher Scientific), and QUANTITY ONE software was used to scan the gray value and determine the target protein expression level relative to that of vinculin.

### Cell proliferation and migration assays

Colony formation tests and the Cell Counting Kit-8 (CCK-8) were used to examine the capacity of cancer cells in various groups to proliferate. Each well of a 96-well plate was filled with 2,500 cancer cells for the CCK-8 experiment. After incubation at 37°C for two hours, 100 µl of CCK-8 solution was added to each well of the 96-well plates (Beijing Solarbio Science & Technology Co., Ltd, Beijing, China), and the absorbance of each well was measured at 450 nm. Briefly, one thousand cells in a cell suspension were seeded in each well of a 6-well plate. After 12-15 days, the cells were fixed with 3.7% formaldehyde (Sigma−Aldrich) and stained with 0.4% crystal violet (Sigma−Aldrich). Each plate was thoroughly washed 3 times in pure water and then scanned. For colony formation experiments, a six-well plate with 1,000 cells in each well was used. Every 72 hours, the culture medium was replaced. When colonies could be seen, crystal violet and 4% paraformaldehyde were used to stain and fix the cells. Transwell assays were conducted to explore the cellular migration ability.

### Statistical analysis

The DEG analysis, univariate and multivariate Cox regression analyses, LASSO regression analysis, correlation analysis, clinicopathological factor analysis, ROC curve analysis, and K-M survival analysis were carried out using R studio (version 4.1.0), GraphPad Prism 8 (version 8.0.1) and R software (version 3.8.0). One-way analysis of variance (ANOVA) and a two-tailed Student’s t test were applied to analyze the data. Statistical significance of differences was set at a P value <0.05.

## Results

### Expression level of CCP-related genes in cutaneous melanoma

**Figure 1:**
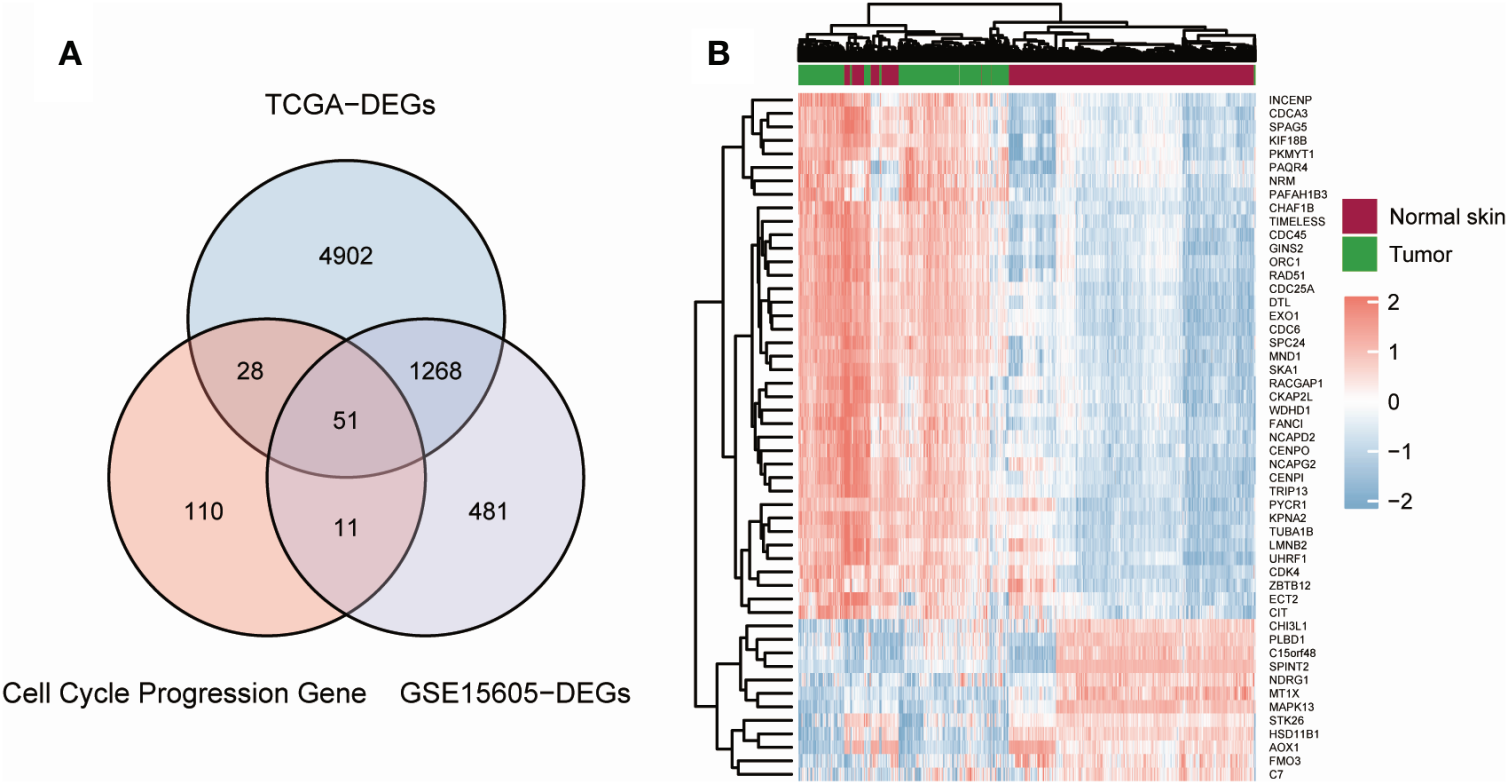
Screening out CCP-related DEGs for construction of a risk signature. **(A)** Venn plot of the intersection of three dataset. **(B)**. Heatmap of CCP-related DEGs in TCGA-GTEx cohort.

First, the limma program was used to examine the DEGs in normal and tumor samples in the TCGA-GTEx (812 normal skin and 469 cutaneous melanoma) and GSE15605 (16 normal skin and 58 cutaneous melanoma) datasets. Comparing the two groups, a total of 6249 DEGs from TCGA-GTEx and 1811 DEGs from GSE15605 were found to be statistically significant (adjusted p value <0.05 and absolute log2-fold change > 1.5). CCP- related genes were integrated from the MSigDB database (**Supplement Table 1**). After that, we combined the three differential gene sets and identified 51 common DEGs (**Figure 1A**). A heatmap displaying information on the 51 genes with the greatest differences in the TCGA-GTEx cohort was then constructed (**Figure 1B**) (**Supplement Table 2**).

### GO analysis and KEGG analysis of CCP-related genes

We conducted an enrichment analysis of these 51 genes to investigate the molecular processes of cutaneous melanoma CCP-related genes. The 51 genes have significant involvement in DNA replication and positive regulation of cell cycle processes, chromosomal area, and condensed chromosomes, according to GO analysis results. The 51 genes have significant functions in the cell cycle, according to KEGG enrichment findings (**Supplement Figures 1A, B**).

### Construction of a CCP-related prognostic signature in cutaneous melanoma

**Figure 2:**
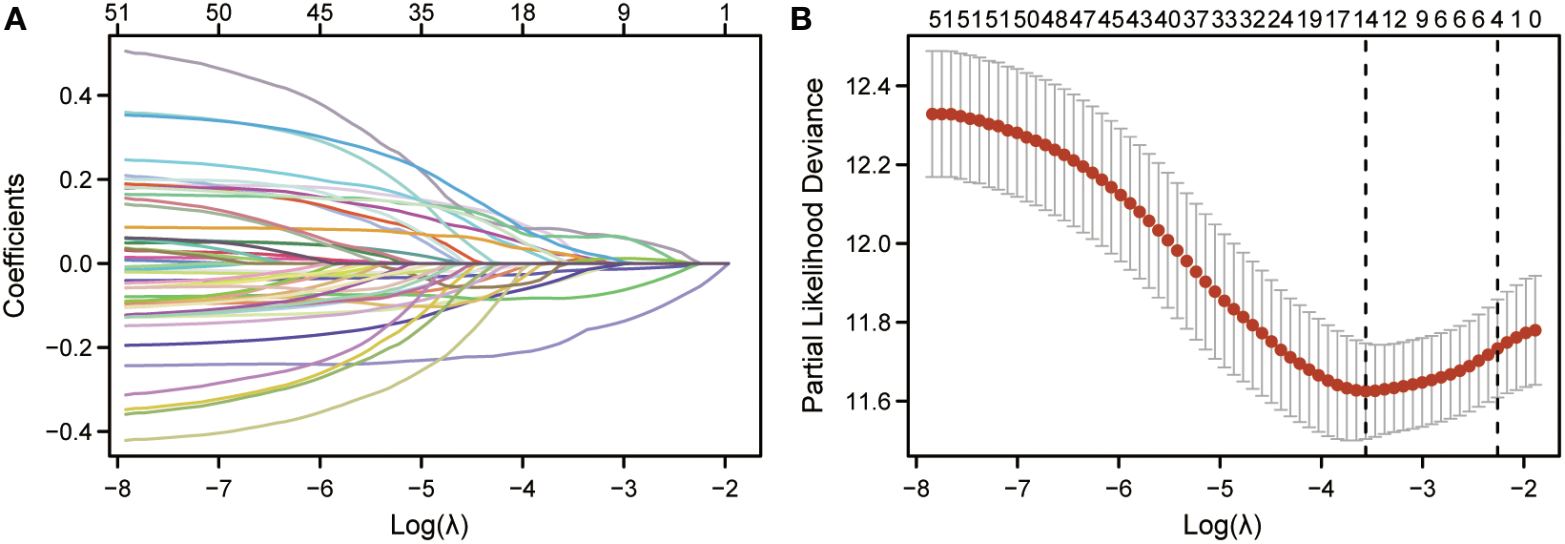
Identification of CCP-related genes in cutaneous melanoma and their biological functions. **(A)**. LASSO coefficient profiles of 51 prognostic CCP-related genes. **(B)**. Cross-validation for tuning parameter selection in the LASSO model.

LASSO regression analysis was further performed on the CCP-related genes to avoid overfitting problems and construct a risk signature, and four genes (HSD11B1, NDRG1, CDCA3 and CHI3L1) were finally screened out according to the optimal lambda value (**Figures 2A, B**). In addition, we performed a multifactorial COX regression analysis of the four genes in the risk-based scoring using the following formula: Riskscore = (-0.2066) * HSD11B1 + (-0.1294) * NDRG1 + (0.2203) * CDCA3). Next, we calculated the riskscore of patients with cutaneous melanoma, and all patients were divided into low- and high-risk groups. The results of multifactorial Cox regression showed that among the four genes, HSD11B1 (HR=0.813, 95%CI=0.715-0.926, P=0.002), NDRG1 (HR=0.879, 95%CI=0.804-0.960, P=0.004) and CDCA3 (HR=1.246, 95%CI=1.038-1.497, P=0.018) were independent prognostic factors (**Table 1**).

**Table 1:** Multi-factor COX regression analysis showing HSD11B1, NDRG1, and CDCA3 as independent prognostic factors.

Characteristics	Total(N)	Univariate analysis			Multivariate analysis	
		Hazard ratio (95% CI)	P value		Hazard ratio (95% CI)	P value
HSD11B1	453	0.781 (0.702-0.869)	**<0.001**		0.813 (0.715-0.926)	**0.002**
NDRG1	453	0.854 (0.783-0.932)	**<0.001**		0.879 (0.804-0.960)	**0.004**
CDCA3	453	1.362 (1.133-1.636)	**<0.001**		1.246 (1.038-1.497)	**0.018**
CHI3L1	453	0.877 (0.823-0.935)	**<0.001**		0.969 (0.896-1.048)	0.431

### Construction and validation of the risk signature based on CCP-related genes for OS

**Figure 3:**
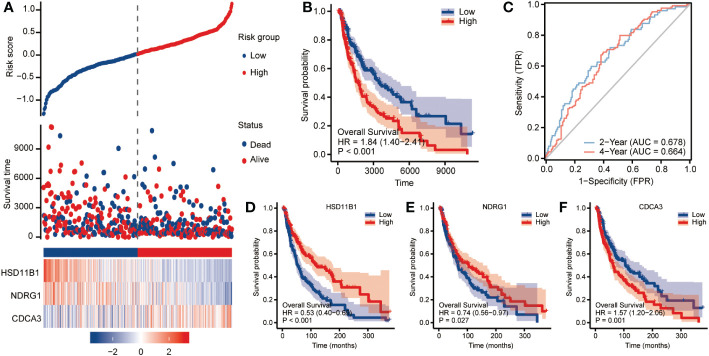
Clinical relevance of CCP-related genes in cutaneous melanoma patients in TCGA. To assess OS outcome, **(A)** distribution of risk score, survival status and the expression of prognostic CCP-related genes; **(B)** Kaplan−Meier plot of the CCP-related gene signature and overall survival; and **(C)** ROC curves for 2- and 4-year survival prediction. Kaplan−Meier plot for the expression of **(D)** HSD11B1, **(E)** NDRG1 and **(F)** CDCA3. The hazard ratios (HRs) were evaluated using Cox proportional hazard models. OS: overall survival; ROC curve: receiver operating characteristic curve.

The cohort served as a validation cohort to assess the predictive effectiveness of the CCP-related gene signature. The patients in datasets were split into high-risk and low-risk groups using the same criteria by computing the risk scores for each patient using the algorithm indicated above. In the high-risk category, there were more fatal cases. High-risk patients consistently exhibited increased expression of HSD11B1 and NDRG1 and reduced expression of CDCA3 (**Figure 3A**). The distribution of this risk model in KM survival curves showed a significantly lower survival status for high-risk patients than for patients in the low-risk group (**Figure 3B**; HR=1.84, 95%CI=1.40-2.41, p<0.001). AUC values (AUC=0.678, 0.664) were found for the ROC curves of this risk model at two and four years, as shown in **Figure 3C**. For HSD11B1 (HR=0.53, 95%CI=0.40-0.69, P<0.001) and NDRG1 (HR=0.74, 95%CI=0.56-0.97, P=0.027), we found that low-RS patients had worse clinical outcomes than high-RS patients, however CDCA3 (HR=1.57, 95%CI=1.20-2.06, p=0.001) exhibited the exact opposite tendency (**Figures 3D-F**). DSS analysis produced the same results (**Supplement Figure 2**).

**Figure 4:**
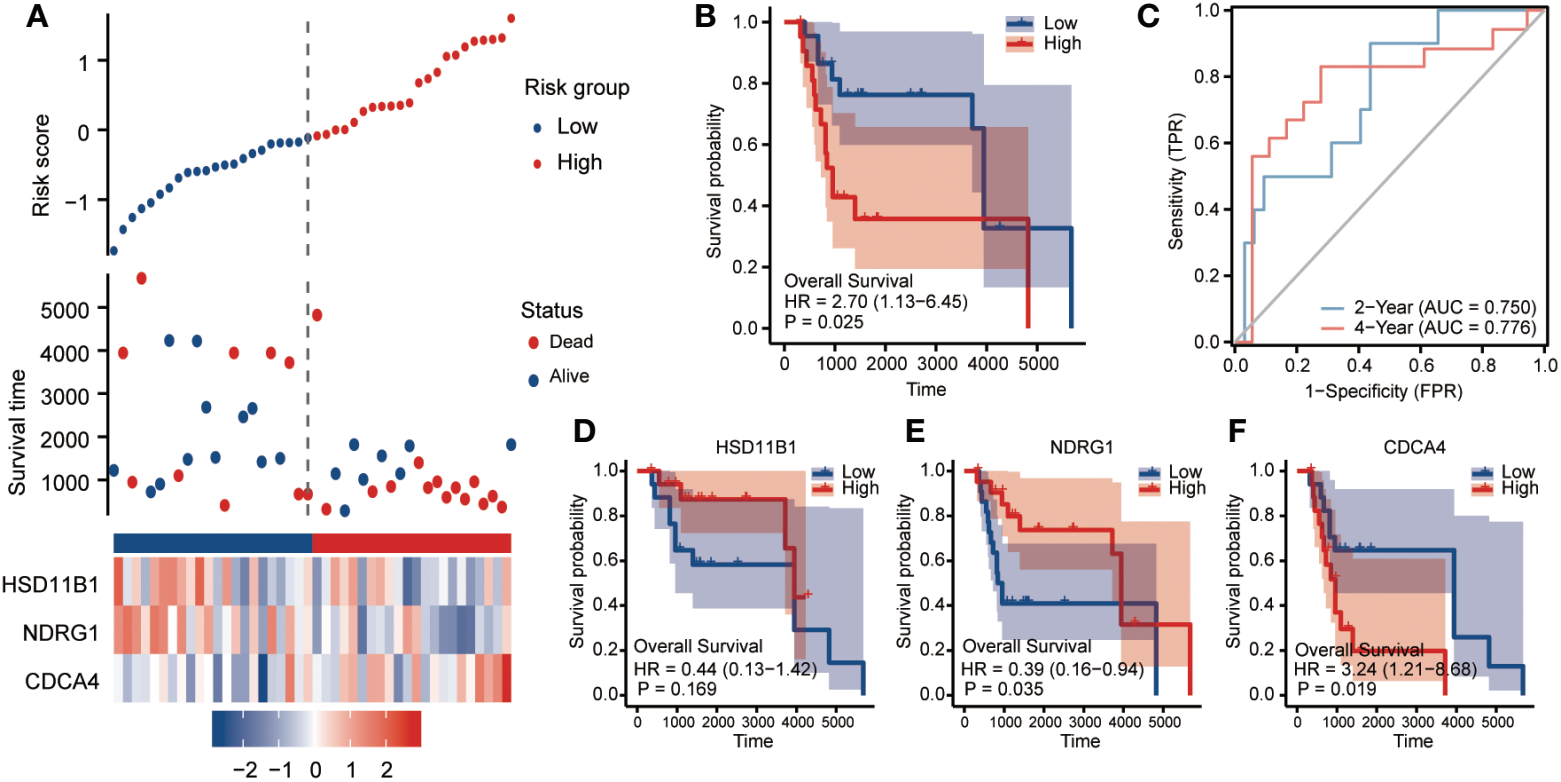
Clinical relevance of CCP-related genes in cutaneous melanoma patients in GSE19234. To assess OS outcome, **(A)** distribution of risk score, survival status and the expression of prognostic CCP-related genes; **(B)** Kaplan−Meier plot of the CCP-related gene signature and overall survival; and **(C)** ROC curves for 2- and 4-year survival prediction. Kaplan−Meier plot for the expression of **(D)** HSD11B1, **(E)** NDRG1 and **(F)** CDCA3. The hazard ratios (HRs) were evaluated using Cox proportional hazard models. OS, overall survival; ROC curve: receiver operating characteristic curve.

The risk signature in the GSE19234 dataset (n = 44) was then externally validated. Patients with cutaneous melanoma were divided into two groups based on the median RS. Patients in the high-risk group had considerably worse overall survival (OS) than those in the low-risk group, which was in line with the indings in the TCGA dataset (**Figures 4A, B** HR=2.7, 95%CI=1.13-6.45, p=0.025). In the testing set, the AUCs of the ROC curves for predicting the 2- and 4-year survival of PC patients were 0.75 and 0.776, respectively (**Figure 4C**), We observed that individuals with a low RS had poorer clinical outcomes than those with a high RS for HSD11B1 (HR=0.44, 95%CI=0.13-1.42, P=0.169) and NDRG1 (HR=0.39, 95%CI=0.16-0.94, P=0.035). However, CDCA3 (HR=3.24, 95%CI=1.21-8.68, P=0.019) displayed the exact opposite tendency, which was consistent with TCGA set results (**Figures 4D-F**).

These findings demonstrate that the gene profile established from CCP-related genes can serve as an accurate prognostic predictor separate from genetic mutation. The above results indicate that the CCP-related RS performs well in terms of cutaneous melanoma prognosis.

### Establishment of a nomogram for cutaneous melanoma OS prognosis

**Figure 5:**
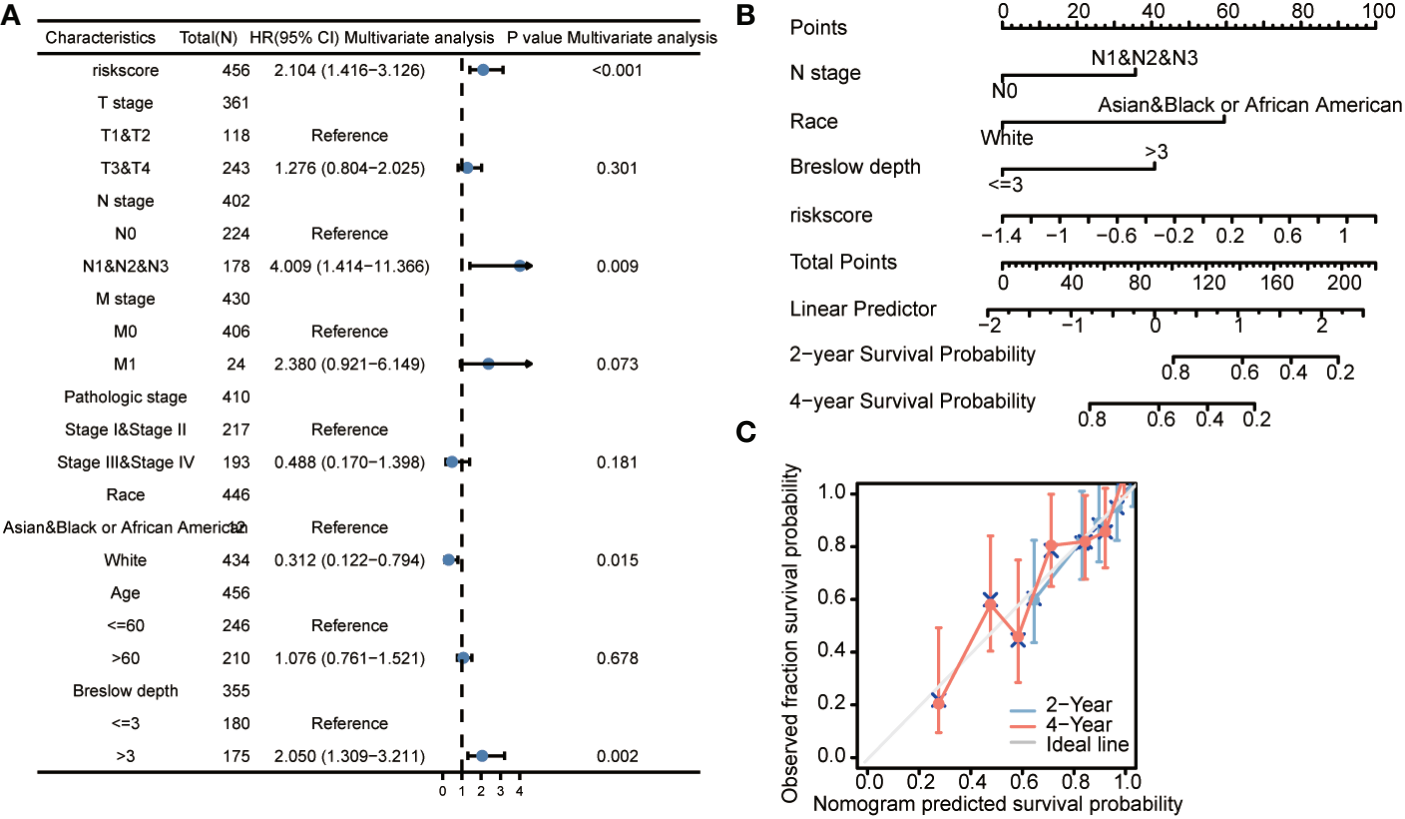
The prognostic value of CCP-related genes (overall survival) in diverse cutaneous melanoma **(A)**. Forrest plot of multivariate Cox regression analysis. **(B)**. Nomogram integrating risk score based on 3 CCP-related genes, N stage, race, and Breslow depth. **(C)**. Calibration plots of the nomogram for evaluating the probability of OS at 2 and 4 years. OS, overall survival.

As shown in multivariate Cox regression models, we analyzed the influence of CCP-related genes on prognosis (OS and DSS) in subgroups. Patients with higher expression of CCP-related genes demonstrated poor OS in the N stage N1&N2&N3 subgroup (HR: 4.009; CI: 1.414–11.366; P = 0.009), race white subgroup (HR: 0.312; CI: 0.122–0.794; P = 0.015), and Breslow depth >3 subgroup (HR: 2.05; CI: 1.309–3.211; P = 0.002) (**Figure 5A**). We developed a nomogram based on independent clinicopathologic variables and CCP-related genes to more accurately predict the survival rates of cutaneous melanoma patients (**Figure 5B**). The outcomes of multivariate Cox regression were used to assign points to each variable using the nomogram point scale. The points for each variable were totalled together, and total scores were determined using the modified range of 1 to 100. The likely prognosis of each cutaneous melanoma patient at 2 and 4 years was established by drawing a direct line from the total score line to the outcome line. For example, a cutaneous melanoma patient with CCP-related genes-high risk (80 points), N stage (0 points), race (0 points) and Breslow depth (40 points) could attain 120 total points. The 2- and 4-year survival rates were approximately 70 and 40%, respectively. Additionally, the nomogram’s effectiveness was assessed, and the calibration curve using a Hosmer test of the nomogram in the TCGA-LIHC cohort was 0.761, indicating that the nomogram had a reasonably accurate prediction efficiency (**Figure 5C**). Increased expression of CCP-related genes was associated with a lower survival rate in subgroups of other variables, according to the results of the DSS subgroup analysis (**Supplement Figure 3**).

### Correlation of the expression of CCP-related genes and clinicopathological features of cutaneous melanoma patients

**Figure 6:**
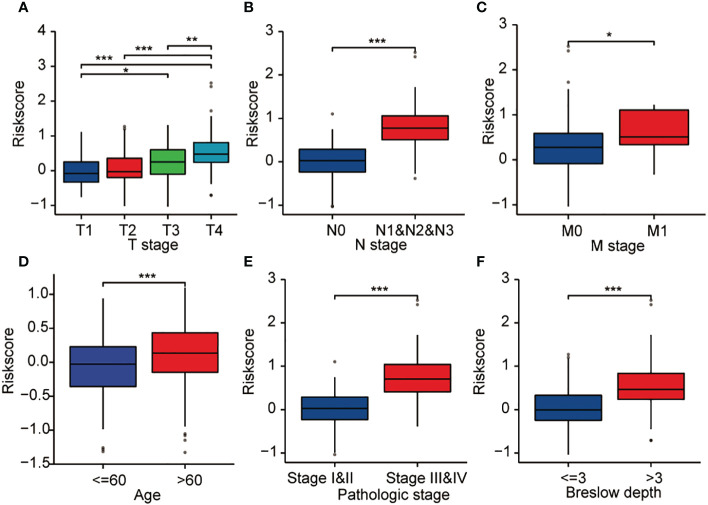
Correlation between CCP-related gene expression and clinicopathological characteristics, including **(A)** T stage, **(B)** N stage, **(C)** M stage, **(D)** age, **(E)** pathologic stage, and **(F)** Breslow depth, in cutaneous melanoma patients in the TCGA cohort. *p < 0.05; **p < 0.01; ***p < 0.001; NS, no significance.

Concurrently, additional studies were conducted on the relationship between patient clinicopathological data and the expression of CCP-related genes. As demonstrated in **Figures 6A-F**, higher expression level of CCP-related genes was significantly related to T stage (T1 vs. T4, P < 0.001), N stage (N0 vs. N1&N2&N3, P < 0.001), M stage (M0 vs. M1, P < 0.05), age (<=60 vs. >60, P < 0.001), pathologic stage (stageI&II vs. stageIII&IV, P < 0.001), and Breslow depth (<=3 vs. >3, P < 0.001) (**Figure 6**).

### CDCA3 is highly expressed and correlated with an unfavorable prognosis in cutaneous melanoma

Due to its high proportion in the risk signature, we tended to consider that CDCA3 occupied the core position in the risk signature. A pancancer analysis of CDCA3 was performed, showing that cutaneous melanoma experienced one of the most remarkably increases in CDCA3 expression among all cancer types (**Supplement Figure 4A**). To be specific, a joint analysis of TCGA and GTEx and GSE15605 databases confirmed that the expression of CDCA3 in cutaneous melanoma tissues was significantly higher than that in normal tissues (**Supplement Figure 4B, C**). Furthermore, we constructed receiver operating characteristic (ROC) curves, and the area under the curve (AUC) for CDCA3 was 0.867, which indicates that CDCA3 is significantly differentially expressed in tumor and normal tissue (**Supplement Figure 4D**). Kaplan−Meier analysis of overall survival, disease-specific survival and progression-free interval data revealed that patients with high CDCA3 expression suffered a poorer prognosis than those with low CDCA3 expression (**Supplement Figure 5A-C**). Then, based on the OS information, we performed subgroup analyses of prognosis, which demonstrated that the survival rates of patients with cutaneous melanoma with higher CDCA3 expression was poor in the T stages I–II, N0, M0, race of white, age > 60, female, Breslow depth< 3 and pathologic stage I−II subgroups (**Supplement Figure 5D-K**).

### Potential mechanism by which CDCA3 regulates cutaneous melanoma progression

We compared 236 cutaneous melanoma CDCA3-high samples with 236 CDCA3-low samples. Between the two groups, a total of 228 DEGs, including 166 downregulated genes and 62 upregulated genes, were found to be significantly differentially expressed (adjusted p value < 0.05 and absolute Log2-fold change > 1.5).

To better analyze the enrichment of the biological function of CDCA3-associated genes, we applied Metascape to explore GO enrichment, which demonstrated that CDCA3-associated genes were involved in a number of biological functions. For instance, differential CDCA3 expression can modulate skin development, epidermal cell differentiation, epithelial cell proliferation, and keratinocyte development. Moreover, antigen binding, immunoglobulin receptor binding and immunoglobulin complex also showed a relationship with CDCA3-related genes (**Supplement Figures 6A, B**).

**Figure 7:**
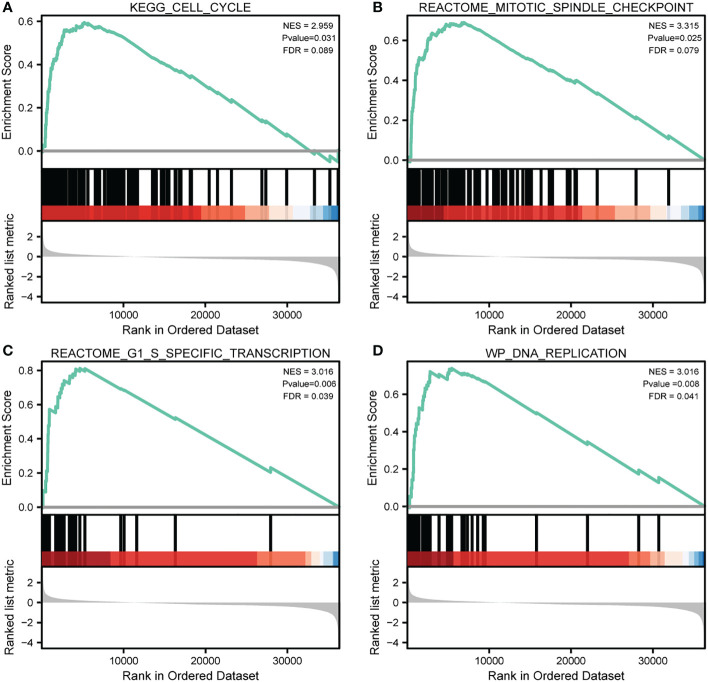
|Significantly enriched annotations of CDCA3-related genes in cutaneous melanoma. **(A–D)** Enrichment plots from the gene set enrichment analysis (GSEA). Several pathways and biological processes were differentially enriched in CDCA3-related cutaneous melanoma, including cell cycle, mitotic spindle checkpoint, G1-S specific transcription, and DNA replication. NES, normalized enrichment score; p. adj, adjusted P value; FDR, false discovery rate.

To evaluate significant differences in the enrichment in MSigDB, we used GSEA to compare the CDCA3-high and -low expression data. In accordance with their normalized enrichment score, we selected the top substantially enriched pathways (NES). GSEA enrichment plots revealed that cell cycle (NES=2.959, P value=0.031), mitotic spindle checkpoint (NES=3.315, P value=0.025), G1-S specific transcription (NES=3.016, P value=0.006), and DNA replication (NES=3.016, P.adj=0.008) were significantly enriched in patients with CCP-related genes (**Figures 7A-D**).

### CDCA3 predicts immune cell infiltration into the cutaneous melanoma microenvironment

**Figure 8:**
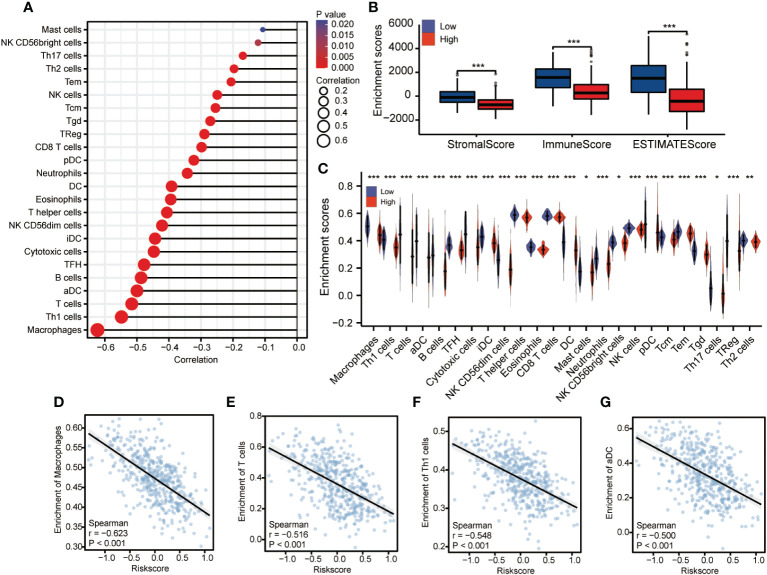
Correlation analysis of the expression of CDCA3 and immune cell infiltration. **(A)** The abundance ratio of the 22 types of immune cells in the entire TCGA set. **(B)** Differences in immune scores between high and low CDCA3 expression groups. **(C)** Differential immune cell type abundance between high and low CDCA3 expression groups. **(D–G)** Correlation diagrams and scatter plots indicating differences in themacrophage, T cell, Th1 cell, and aDC infiltration levels between the high and low CDCA3 expression groups. *p < 0.05; **p < 0.01; ***p <0.001; NS, no significance.

Next, the CIBERSORT method was used to determine the percentages of 22 different types of immune cells among the total accumulated cells (**Figure 8A**). Additionally, the immunological microenvironment was scored using the ESTIMATE program, which showed that the group with high CDCA3 expression had a considerably lower immune score than the group with low CDCA3 expression (**Figure 8B**). These findings demonstrate that the high CDCA3 expression group had a comparatively greater number of 22 different immune cell types than the group with low CDCA3 expression (**Figure 8C**). As shown in **Figures 8D-G**, the expression of CDCA3 was negatively associated with the abundance of innate immune cells, including macrophages (r = −0.623, P < 0.001), T cells (r = −0.516, P < 0.001), Th1 cells (R = −0.548, P < 0.001), and aDCs (R = −0.5, P < 0.001).

### Knockdown of CDCA3 suppresses the malignant phenotype of cutaneous melanoma *In vitro*

**Figure 9:**
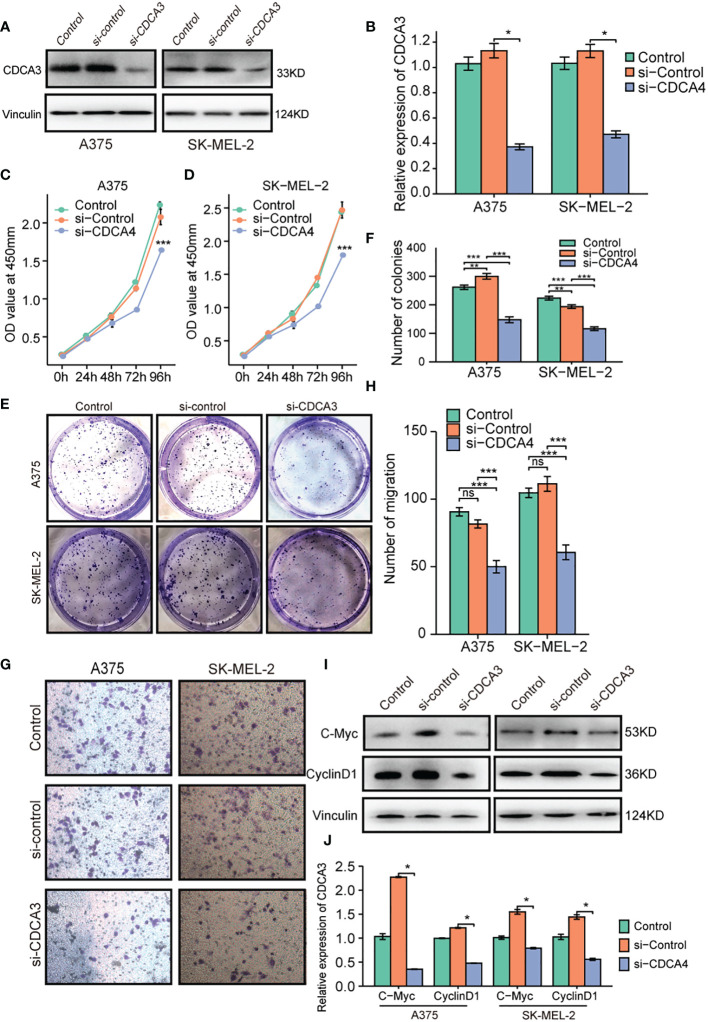
Decreased CDCA3 expression inhibits the proliferation and migration of cutaneous melanoma cells *in vitro*. **(A)** The si-CDCA3 transfection efficiency in A375 and SK-MEL-2 cell lines was explored *via* western blotting. **(B)** Representative statistical analysis of CDCA3 expression in the control, si-control, and si-CDCA3 groups. **(C, D)** CCK-8 assays were applied to detect the effect of CDCA3 knockdown on the proliferation of A375 and SK-MEL-2 cell lines. **(E)** Images of colony formation assay results after CDCA3 knockdown in the A375 and SK-MEL-2 cell lines. **(F)** Representative statistical analysis of the colony formation assay results, including the control, si-control, and si-CDCA3 groups. **(G)** Images of the transwell assay results after CDCA3 knockdown in the A375 and SK-MEL-2 cell lines. **(H)** Representative statistical analysis of the transwell assay results. **(I)** The expression of C-Myc and CyclinD1 was explored by western blotting. **(J)** Representative statistical analysis of C-Myc and CyclinD1 expression in the control, si-control, and si-CDCA3 groups. *p < 0.05; **p < 0.01; ***p < 0.001; NS, no significance.

To gain insight into how CDCA3 affects the course of cutaneous melanoma, we inhibited the expression of CDCA3 in cutaneous melanoma cells by transfecting them with si-CDCA3. CDCA3 was confirmed to be downregulated in A375 and SK-MEL-2 cells transfected with si-CDCA3 compared with the level in cells transfected with si-control (**Figures 9A, B**). CCK-8 assys were used to gauge ‘the effect of CDCA3 on cell proliferation. The OD values for A375 and SK-MEL-2 cells transfected with si-CDCA3 were obviously lower than that of cells transfected with si-control, as shown in **Figures 9C, D**. These findings demonstrate that CDCA3 knockdown significantly reduced cutaneous melanoma cell growth. After that, an investigation of plate colony development was conducted to confirm these findings. The colonies produced by A375 and SK-MEL-2 cells transfected with si-CDCA3 were much smaller than those produced by cells transfected with si-control (**Figures 9E, F**). Collectively, CDCA3 promotes the viability of cutaneous melanoma cells. Transwell experiments revealed that the number of migratory cells in the si-CDCA3 group was much lower than that in A375 and SK-MEL-2 cells transfected with si-control (**Figures 9G, H**). These results indicate that CDCA3 can significantly improve the migration capacity of cutaneous melanoma cells. In addition, we determined the relative expression levels of C-Myc and CyclinD1 because we hypothesized that CDCA3 is involved in the control of cell cycle-related proteins to carry out its biological role in cutaneous melanoma. The findings indicated that suppression of CDCA3 in cutaneous melanoma cells noticeably reduces the expression of C-Myc and CyclinD1 (**Figures 9I, J**). These findings suggest that CDCA3 controls cell cycle-related protein expression in cutaneous melanoma.

## Discussion

Oncological biomarkers with diagnostic, prognostic, and predictive significance will likely become more important in this new era of precision medicine (18). The prevalence of cutaneous melanoma has been increasing yearly. Cutaneous melanoma has a terrible prognosis after it has invaded through the dermis, and the death rate is not expected to decrease in the upcoming years (19). At present, pathological clinical information plays a role in assessing prognosis. Researchers have found that age, histological regression, a larger Breslow thickness, ulceration, neurotropism, and mitogenicity are important predictors of sentinel prognosis in cutaneous melanoma (20–22). In addition, appropriate biomarkers should enable not only monitoring of the disease progression and response to treatment but also the early identification of individuals at high risk of metastases who may benefit from closer surveillance and adjuvant medicines (18, 23). A study reported by Huang and Han et al. primarily identified five chemokine members (CCL4, CCL5, CXCL9, CXCL10, CXCL13) as relevant biomarkers in cutaneous melanoma tumorigenesis and progression (24). Alimohammadi et al. revealed that CXCR4 is implicated in the progression and metastasis of cutaneous melanoma (25). Serum lactate dehydrogenase (LDH) and S100B protein levels are also associated with a poor prognosis and a diminished response to therapy in cutaneous melanoma patients (26). These studies evaluated chemokines and specific proteins as indicators; however, to date, gene-level prognostic markers related to cancer cell division and differentiation, which are most closely associated with tumor growth, are still needed.

In the MSigDB database, 528 CCP-related genes have been identified, but only a small proportion of them have been thoroughly investigated. Recent investigations have shown that CCP-related genes play a significant role in the development of many cancer types *via* a variety of pathways.

The type 1 isoform of 11-hydroxysteroid dehydrogenase, which transforms glucocorticoids into their active form and is crucial for controlling immunological response, cell proliferation, and differentiation, is encoded by the HSD11B1 gene. Numerous studies have investigated the connections between HSD11B1 polymorphisms and human cancer risk in recent years (27–29). According to Feigelson et al., areas of the genome that may contain risk alleles for breast cancer are marked by tagging SNPs in HSD11B1 and IRS2, and these relationships are likely independent of obesity (30). Research conducted to evaluate the connections between HSD11B1 polymorphisms and the cancer risk have thus far produced conflicting findings. The high expression of this gene observed in our study was found to be a positive predictive sign. We hypothesize that this is connected to differences among cancer types and the adaptability brought on by the polymorphism of this gene given the disparate results from the prior research.

N-myc-downregulated gene 1 (NDRG1) interacts with several established tumor signaling pathways; has significant anticancer properties; and prevents cell growth, survival, metastasis, and angiogenesis. According to the findings of Lim et al., NDRG1 plays a crucial role in androgen signaling and has the potential to serve as a major therapeutic target and biomarker in prostate cancer (31). According to studies by Chang et al., DNMT family DNA methylation may be the cause of the downregulation of NDRG1 expression in gastric cancer. Patients with stomach cancer may benefit from using a demethylating agent as a targeted medication (32). NDRG1 has significant scientific implications in both prostate cancer and glioblastoma (33, 34).

Cell division cycle-associated gene 3, also known as trigger of mitotic entry 1 (TOME-1), was initially discovered as a modulator of cell cycle progression for entrance into mitosis from the G2 phase (13). The cell cycle is regulated by a gene family related to cell division. Numerous studies have shown that CDCA gene malfunction can affect immune cell infiltration in tumors and unchecked cell proliferation that results in carcinogenesis (14). There has also been evidence of increased CDCA3 expression in many cancer types. High CDCA3 mRNA expression is substantially linked with several clinicopathologic characteristics in individuals with renal papillary kidney disease, according to Li et al. High CDCA3 mRNA expression has been reported to be related to shorter overall survival, progression-free interval, and disease-specific survival (35). Similar results were discovered for malignancies of the lung, prostate, and nasopharynx (14–16). Overall, CDCA3 is an effective prognostic marker and a promising target for the development of novel anticancer therapies.

Previous studies have described different molecular markers or gene expression profiles with independent prognostic significance for the prediction of survival or metastasis risk associated with cutaneous melanoma (5–7). However, meta-analysis findings have shown that the prognostic ability of DecisionDx-Melanoma and MelaGenix varies by American Joint Committee on Cancer stage (36). To conduct more in-depth research and develop an accurate prediction model, we screened the gene signature of cutaneous melanoma compared with normal skin *via* bioinformatics analysis. Due to the close correlation between CCP-related genes and tumors, the intersection of CCP-related genes and cutaneous melanoma gene signatures was selected for risk assessment and prognostic analysis, and the most representative gene was singled out and verified *in vitro*.

In our study, CCP-related genes were found to be a major risk factor for the prognosis of cutaneous melanoma patients. Effective bioinformatic techniques were used for accurate candidate variable screening and to develop a unique gene signature generated from CCP-related genes to assess the prognosis of patients with cutaneous melanoma. We first subjected the differentially expressed genes to enrichment analysis, and the results showed that the gene functions are focused on DNA replication and positive regulation of cell cycle processes, chromosomal area, and condensed chromosomes, according to the results of the GO and KEGG analyses. In terms of controlling tumor progression, cell cycle is a critical regulatory step to inhibit the unlimited proliferation of tumor cells. Then, we used the LASSO approach to create a reliable gene signature for cutaneous melanoma prognosis generated from CCP-related genes. The three CCP-related gene signatures–HSD11B1, NDRG1 and CDCA3–may each independently predict the clinical outcomes of cutaneous melanoma patients, according to our prognostic study (both in OS and DSS). Patients who had high RS had a poor prognosis. ROC curves for 2- and 4-year survival had a relatively high ROC score and supported the accuracy of the prediction. The external validation demonstrated the clinical usefulness of this gene signature produced from CCP-related genes. We created a nomogram by combining stage and the CCP-related gene signature to provide a personalized score for respective factors. After ROC, calibration, and conclusive curves were confirmed, this nomogram showed promise as a clinical tool for cutaneous melanoma prognosis prediction in the future.

Previous studies have demonstrated that there are diverse associations between clinical pathological factors and prognosis in cutaneous melanoma (8, 20–22). According to the findings of our investigation, high levels of CCP-related gene expression are associated with advanced clinical pathologic features and a poor prognosis in cutaneous melanoma. In a stratified analysis, we discovered that the expression of CCP-related genes was a significant predictor of prognosis for specific subgroups, including T1 and T2 vs. T3 and T4 stage, N0 vs. N1, N2 and N3 stages, M0 vs. M1 stage, pathologic stage I and II vs. stages III and IV, and in different Breslow depth groups, showing that CCP-related genes are independent of these important clinicopathological criteria.

Recent studies have demonstrated that tumor-infiltrating immune cells can influence how a tumor develops and progresses (37). Our results indicated that CCP-related gene expression in cutaneous melanoma is negatively associated with infiltration of multiple types of immune cells, such as macrophages, T cells, Th1 cells and aDCs, which have a significant impact on the initiation and regulation of cancer immune responses. Our findings confirmed previous results that describe functional correlations of melanoma-specific CD4^+^ T cells, macrophages and dendritic cells with cancer phenotypes, suggesting that these cells may be used to alter the tumor microenvironment (38, 39). Previous studies have also demonstrated increased T helper 1 and Type 1 CD8+ T-cell activity, suggesting that the enhanced antitumor efficacy of combination immunotherapy is primarily due to an increase in CD8+ T-cell capacity to mediate antitumor cellular immunity (40).

Given that CDCA3 accounted for a large fraction of the risk signature and held the most important position in the analysis of DEGs based on the risk signature, we tend to believe that CDCA3 holds the central position in the risk signature. Therefore, in the subsequent investigation, we focused on CDCA3. As shown by the GSEA results, high levels of CCP-related gene expression were shown by GSEA to trigger DNA replication, the G1-S specific transcription factor, the mitotic spindle checkpoint, and cell cycle progression, which demonstrates that CDCA3 is a regulator of tumor cell cycle progression. The effects of CDCA3 on cutaneous melanoma cell proliferation and migration were subsequently evaluated. As anticipated, we discovered that knocking down CDCA3 decreased the malignant phenotype of cutaneous melanoma cells, verifying that CDCA3 expression is a promising target for cutaneous melanoma treatment. The underlying processes of CDCA3 in cutaneous melanoma should be clarified in further investigations. C-Myc and CyclinD1 are involved in cell cycle regulation, which might encourage metastasis of tumor cells (41). In the present study, western blot analysis was used to determine the relationship between CDCA3 and the expression of cell cycle protein markers. The results showed that CDCA3 suppression might decrease C-Myc and CyclinD1 expression levels. These findings indicate that CDCA3 affects cutaneous melanoma *via* cell cycle protein regulation.

Our investigation has various limitations. First, in cutaneous melanoma samples from TCGA and GEO, the differentiation of samples with or without resistance to targeted therapy and immune therapy was not considered, nor were BRAF/NRAS mutations in patient tumors. Second, although three genes (HSD11B1, NDRG1 and CDCA3) showed a robust prognostic effect based on validation of the expression of screened prognostic genes in TCGA and GEO datasets, regrettably, some other upstream and downstream signals with predictive values were not considered in this study, and further biological evidence—such as animal experimentation—is required given that the prognostic signature was developed and verified only by utilizing data from open databases.

## Conclusion

Despite recent advances in innovative targeted medicines and immunotherapy, ensuring a good prognosis remains an unfulfilled goal. As a result, developing improved treatment targets and prognostic biomarkers is an important objective in cutaneous melanoma research. In our study, the progression of cutaneous melanoma was discovered to be closely correlated with cell division cycle-associated gene 3, which may contribute to the proper evaluation of patient prognosis and an improvement in clinical decision-making.

## Data availability statement

The original contributions presented in the study are included in the article/**Supplementary Material**. Further inquiries can be directed to the corresponding authors.

## Ethics statement

This study, which involved human participants, was examined and accepted for approval by the Peking Union Medical College. The patients/participants in this research provided written informed consent to participate in this research.

## Author contributions

TL and LW performed all experiments and data analyses and wrote the original manuscript. NY, JH and AZ wrote and critically reviewed the manuscript. XL conceived, designed, and directed the study. All authors contributed to the article and approved the submitted version.

## Supplementary material

Supplementary Figure 1 Identification of CCP-related genes in cutaneous melanoma and their biological functions. **(A)**. Histogram showing the significant biological process, cellular component, molecular function, and KEGG pathways enriched by CCP-related genes. **(B)**. Networks showing the significant biological process, cellular component, molecular function, and KEGG pathways enriched by CCP-related genes.

Supplementary Figure 2 Clinical relevance of CCP-related genes in patients with cutaneous melanoma in TCGA. To assess DSS outcome, **(A)** distribution of risk score, survival status and the expression of prognostic CCP-related genes; **(B)** Kaplan−Meier plot of the CCP-related gene signature and overall survival; and **(C)** ROC curves for 2- and 4-year survival prediction. Kaplan−Meier plot for the expression of **(D)** HSD11B1, (E) NDRG1 and (F) CDCA3. The hazard ratios (HRs) were evaluated using Cox proportional hazard models. OS: overall survival; ROC curve: receiver operating characteristic curve.

Supplementary Figure 3 The prognostic value of CCP-related genes (disease-specific survival) in diverse cutaneous melanoma **(A)**. Forrest plot showing the results of multivariate Cox regression analysis. **(B)**. Nomogram integrating the 3 CCP-related gene-based risk score, N stage, race, and Breslow depth. **(C)**. Calibration plots of the nomogram for evaluating the probability of OS at 2 and 4 years. OS, overall survival.

Supplementary Figure 4 The expression levels of CDCA3 in different cancer types and in cutaneous melanoma. **(A)** Increased or decreased CDCA3 expression in different cancer types compared with normal tissues in TCGA and GTEx databases. **(B–D)** The expression levels of CDCA3 in cutaneous melanoma. **(E)** ROC curve to investigate the value of CDCA3 in identifying cutaneous melanoma tissues.

Supplementary Figure 5 Kaplan–Meier survival plots comparing the low and high expression of CDCA3 in cutaneous melanoma. **(A–C)** Survival curves of OS, DSS, and PFI between high and low CDCA3 expression in patients with cutaneous melanoma. **(D–K)** OS survival curves of T stages I–II, N0, M0, pathologic stage I−II, race of white, age > 60, female, Breslow depth< 3 subgroups between high and low CDCA3 expression in patients with cutaneous melanoma. OS, overall survival; DSS, disease-specific survival; PFI, progression-free interval.

Supplementary Figure 6 Functional enrichment of CDCA3 in cutaneous melanoma. **(A)** Volcano plots of the DEGs between high and low CDCA3 expression in cutaneous melanoma. **(B)** Significantly enriched GO annotations of CDCA3-related genes in cutaneous melanoma.

## References

[1] Tripp MK Watson M Balk SJ Swetter SM Gershenwald JE. State of the science on prevention and screening to reduce melanoma incidence and mortality: The time is now. CA: Cancer J Clin (2016) 66(6):460–80. doi: 10.3322/caac.21352 PMC5124531 27232110

[2] Johansson I Tempel D Dwarkasing JT Rentroia-Pacheco B Mattsson J Ny L. Validation of a clinicopathological and gene expression profile model to identify patients with cutaneous melanoma where sentinel lymph node biopsy is unnecessary. Eur J Surg Oncol (2022) 48(2):320–5. doi: 10.1016/j.ejso.2021.11.010 34794843

[3] Alderman C Sehlaoui A Xiao Z Yang Y. MicroRNA-15a inhibits the growth and invasiveness of malignant melanoma and directly targets on CDCA4 gene. Tumour Biol (2016) 37(10):13941–50. doi: 10.1007/s13277-016-5271-z 27492455

[4] Trager MH Geskin LJ Samie FH Liu L. Biomarkers in melanoma and non-melanoma skin cancer prevention and risk stratification. Exp Dermatol (2022) 31(1):4–12. doi: 10.1111/exd.14114 32415889

[5] Kashani-Sabet M Venna S Nosrati M Rangel J Sucker A Egberts F. A multimarker prognostic assay for primary cutaneous melanoma. Clin Cancer Res (2009) 15(22):6987–92. doi: 10.1158/1078-0432.CCR-09-1777 PMC2784204 19887476

[6] Gerami P Cook RW Wilkinson J Russell MC Dhillon N Amaria RN. Development of a prognostic genetic signature to predict the metastatic risk associated with cutaneous melanoma. Clin Cancer Res (2015) 21(1):175–83. doi: 10.1158/1078-0432.CCR-13-3316 25564571

[7] Garg M Couturier DL Nsengimana J Fonseca NA Wongchenko M Yan Y. Tumour gene expression signature in primary melanoma predicts long-term outcomes. Nat Commun (2021) 12(1):1137. doi: 10.1038/s41467-021-21207-2 33602918 PMC7893180

[8] Hsu CC Lee TL Lin MH Liao YH Liau JY Sheen YS. Risk factors for lymphatic and hematogenous metastasis after diagnosis of cutaneous melanoma in Taiwan. J Formos Med Assoc (2022). doi: 10.1016/j.jfma.2022.02.018 35292188

[9] Matthews HK Bertoli C de Bruin RAM. Cell cycle control in cancer. Nat Rev Mol Cell Biol (2022) 23(1):74–88. doi: 10.1038/s41580-021-00404-3 34508254

[10] Evan GI Vousden KH. Proliferation, cell cycle and apoptosis in cancer. Nature (2001) 411(6835):342–8. doi: 10.1038/35077213 11357141

[11] Cooperberg MR Simko JP Cowan JE Reid JE Djalilvand A Bhatnagar S. Validation of a cell-cycle progression gene panel to improve risk stratification in a contemporary prostatectomy cohort. J Clin Oncol (2013) 31(11):1428–34. doi: 10.1200/JCO.2012.46.4396 23460710

[12] Hui Y Leng J Jin D Liu D Wang G Wang Q. A cell cycle progression-derived gene signature to predict prognosis and therapeutic response in hepatocellular carcinoma. Dis Markers (2021) 2021:1986159. doi: 10.1155/2021/1986159 34721731 PMC8553501

[13] Yoshida K. Cell-cycle-dependent regulation of the human and mouse tome-1 promoters. FEBS Lett (2005) 579(6):1488–92. doi: 10.1016/j.febslet.2005.01.055 15733861

[14] Jiang D Li Y Cao J Sheng L Zhu X Xu M. Cell division cycle-associated genes are potential immune regulators in nasopharyngeal carcinoma. Front Oncol (2022) 12:779175. doi: 10.3389/fonc.2022.779175 35237510 PMC8882974

[15] Chen J Zhu S Jiang N Shang Z Quan C Niu Y. HoxB3 promotes prostate cancer cell progression by transactivating CDCA3. Cancer Lett (2013) 330(2):217–24. doi: 10.1016/j.canlet.2012.11.051 23219899

[16] Adams MN Burgess JT He Y Gately K Snell C Zhang SD. Expression of CDCA3 is a prognostic biomarker and potential therapeutic target in non-small cell lung cancer. J Thorac Oncol (2017) 12(7):1071–84. doi: 10.1016/j.jtho.2017.04.018 28487093

[17] Bindea G Mlecnik B Tosolini M Kirilovsky A Waldner M Obenauf AC. Spatiotemporal dynamics of intratumoral immune cells reveal the immune landscape in human cancer. Immunity (2013) 39(4):782–95. doi: 10.1016/j.immuni.2013.10.003 24138885

[18] Ita MI Wang JH Fanning N Kaar G Lim C Redmond HP. Plasma circulating cell free messenger RNA as a potential biomarker of melanoma. Acta Oncol (2021) 60(9):1201–9. doi: 10.1080/0284186X.2021.1928749 34086522

[19] Lebbé C Meyer N Mortier L Marquez-Rodas I Robert C Rutkowski P. Evaluation of two dosing regimens for nivolumab in combination with ipilimumab in patients with advanced melanoma: Results from the phase IIIb/IV CheckMate 511 trial. J Clin Oncol (2019) 37(11):867–75. doi: 10.1200/JCO.18.01998 PMC6455714 30811280

[20] Aivazian K Ahmed T El Sharouni MA Stretch JR Saw RPM Spillane AJ. Histological regression in melanoma: impact on sentinel lymph node status and survival. Mod Pathol (2021) 34(11):1999–2008. doi: 10.1038/s41379-021-00870-2 34247192

[21] Shannon AB Wood C Straker RJ 3rd Miura JT Ming ME Elenitsas R. Age and mitogenicity are important predictors of sentinel lymph node metastasis in T1a melanoma. Ann Surg Oncol (2021) 28(9):4777–9. doi: 10.1245/s10434-021-09929-5 33834324

[22] Walker RJB Look Hong NJ Moncrieff M van Akkooi ACJ Jost E Nessim C. Predictors of sentinel lymph node metastasis in patients with thin melanoma: An international multi-institutional collaboration. Ann Surg Oncol (2022). doi: 10.1245/s10434-022-11936-z 35676603

[23] Broggi MAS Maillat L Clement CC Bordry N Corthesy P Auger A. Tumor-associated factors are enriched in lymphatic exudate compared to plasma in metastatic melanoma patients. J Exp Med (2019) 216(5):1091–107. doi: 10.1084/jem.20181618 PMC6504224 30975896

[24] Si Z Hu H. Identification of CXCL13 as an immune-related biomarker associated with tumorigenesis and prognosis in cutaneous melanoma patients. Med Sci Monit (2021) 27:e932052. doi: 10.12659/MSM.932052 34247183 PMC8280950

[25] Alimohammadi M Rahimi A Faramarzi F Alizadeh-Navaei R Rafiei A. Overexpression of chemokine receptor CXCR4 predicts lymph node metastatic risk in patients with melanoma: A systematic review and meta-analysis. Cytokine (2021) 148:155691. doi: 10.1016/j.cyto.2021.155691 34464923

[26] Buchbinder EI Flaherty KT. Biomarkers in melanoma: Lessons from translational medicine. Trends Cancer (2016) 2(6):305–12. doi: 10.1016/j.trecan.2016.05.003 28741528

[27] Li CF Liu TT Wang JC Yu SC Chen YY Fang FM. Hydroxysteroid 11-beta dehydrogenase 1 overexpression with copy-number gain and missense mutations in primary gastrointestinal stromal tumors. J Clin Med (2018) 7(11). doi: 10.3390/jcm7110408 PMC6262574 30388854

[28] Han D Yu Z Zhang H Liu H Wang B Qian D. Microenvironment-associated gene HSD11B1 may serve as a prognostic biomarker in clear cell renal cell carcinoma: a study based on TCGA, RTqPCR, Western blotting, and immunohistochemistry. Bioengineered (2021) 12(2):10891–904. doi: 10.1080/21655979.2021.1994908 PMC8810109 34845968

[29] Wang J Gao Y Wang L Liu X Li J Wang Z. A variant (rs932335) in the HSD11B1 gene is associated with colorectal cancer in a Chinese population. Eur J Cancer Prev (2013) 22(6):523–8. doi: 10.1097/CEJ.0b013e3283656346 24061267

[30] Feigelson HS Teras LR Diver WR Tang W Patel AV Stevens VL. Genetic variation in candidate obesity genes ADRB2, ADRB3, GHRL, HSD11B1, IRS1, IRS2, and SHC1 and risk for breast cancer in the cancer prevention study II. Breast Cancer Res (2008) 10(4):R57. doi: 10.1186/bcr2114 18611262 PMC2575528

[31] Lim SC Geleta B Maleki S Richardson DR Kovačević Ž. The metastasis suppressor NDRG1 directly regulates androgen receptor signaling in prostate cancer. J Biol Chem (2021) 297(6):101414. doi: 10.1016/j.jbc.2021.101414 34785213 PMC8668986

[32] Chang X Ma J Xue X Wang G Yan T Su L. DNMT family induces down-regulation of NDRG1 *via* DNA methylation and clinicopathological significance in gastric cancer. PeerJ (2021) 9:e12146. doi: 10.7717/peerj.12146 34616614 PMC8450010

[33] Zhang X Chen Q Li Y Chen H Jiang Q Hu Q. N-myc downstream-regulated gene 1 (NDRG1) regulates vascular endothelial growth factor a (VEGFA) and malignancies in glioblastoma multiforme (GBM). BioMed Res Int (2022) 2022:3233004. doi: 10.1155/2022/3233004 35813230 PMC9262576

[34] Panigrahi SK Broustas CG Cuiper PQ Virk RK Lieberman HB. FOXP1 and NDRG1 act differentially as downstream effectors of RAD9-mediated prostate cancer cell functions. Cell Signal (2021) 86:110091. doi: 10.1016/j.cellsig.2021.110091 34298089 PMC8403642

[35] Li H Li M Yang C Guo F Deng S Li L. Prognostic value of CDCA3 in kidney renal papillary cell carcinoma. Aging (Albany NY) (2021) 13(23):25466–83. doi: 10.18632/aging.203767 PMC8714141 34905505

[36] Marchetti MA Coit DG Dusza SW Yu A McLean L Hu Y. Performance of gene expression profile tests for prognosis in patients with localized cutaneous melanoma: A systematic review and meta-analysis. JAMA Dermatol (2020) 156(9):953–62. doi: 10.1001/jamadermatol.2020.1731 PMC7391179 32745161

[37] Sun H Liu L Huang Q Liu H Huang M Wang J. Accumulation of tumor-infiltrating CD49a(+) NK cells correlates with poor prognosis for human hepatocellular carcinoma. Cancer Immunol Res (2019) 7(9):1535–46. doi: 10.1158/2326-6066.CIR-18-0757 31311791

[38] Veatch JR Lee SM Shasha C Singhi N Szeto JL Moshiri AS. Neoantigen-specific CD4(+) T cells in human melanoma have diverse differentiation states and correlate with CD8(+) T cell, macrophage, and b cell function. Cancer Cell (2022) 40(4):393–409.e9. doi: 10.1016/j.ccell.2022.03.006 35413271 PMC9011147

[39] Fukuda K Okamura K Riding RL Fan X Afshari K Haddadi NS. AIM2 regulates anti-tumor immunity and is a viable therapeutic target for melanoma. J Exp Med (2021) 218(9). doi: 10.1084/jem.20200962 PMC8329870 34325468

[40] Zhang Z Zhou H Liu Y Ren J Wang J Sang Q. Anti-PD1 antibody enhances the anti-tumor efficacy of MUC1-MBP fusion protein vaccine via increasing Th1, Tc1 activity and decreasing the proportion of MDSC in the B16-MUC1 melanoma mouse model. Int Immunopharmacol (2021) 101(Pt A):108173. doi: 10.1016/j.intimp.2021.108173 34607233

[41] Hassan MS Cwidak N Johnson C Däster S Eppenberger-Castori S Awasthi N. Therapeutic potential of the cyclin-dependent kinase inhibitor flavopiridol on c-myc overexpressing esophageal cancer. Front Pharmacol (2021) 12:746385. doi: 10.3389/fphar.2021.746385 34621175 PMC8490822

