# Supplementary figures and images for: CDCA3 is a prognostic biomarker for cutaneous melanoma and is connected with immune infiltration
Source: Front Oncol. 2023 Jan 11;12:1055308. doi: 10.3389/fonc.2022.1055308 (PMC9876620; doi:10.3389/fonc.2022.1055308)

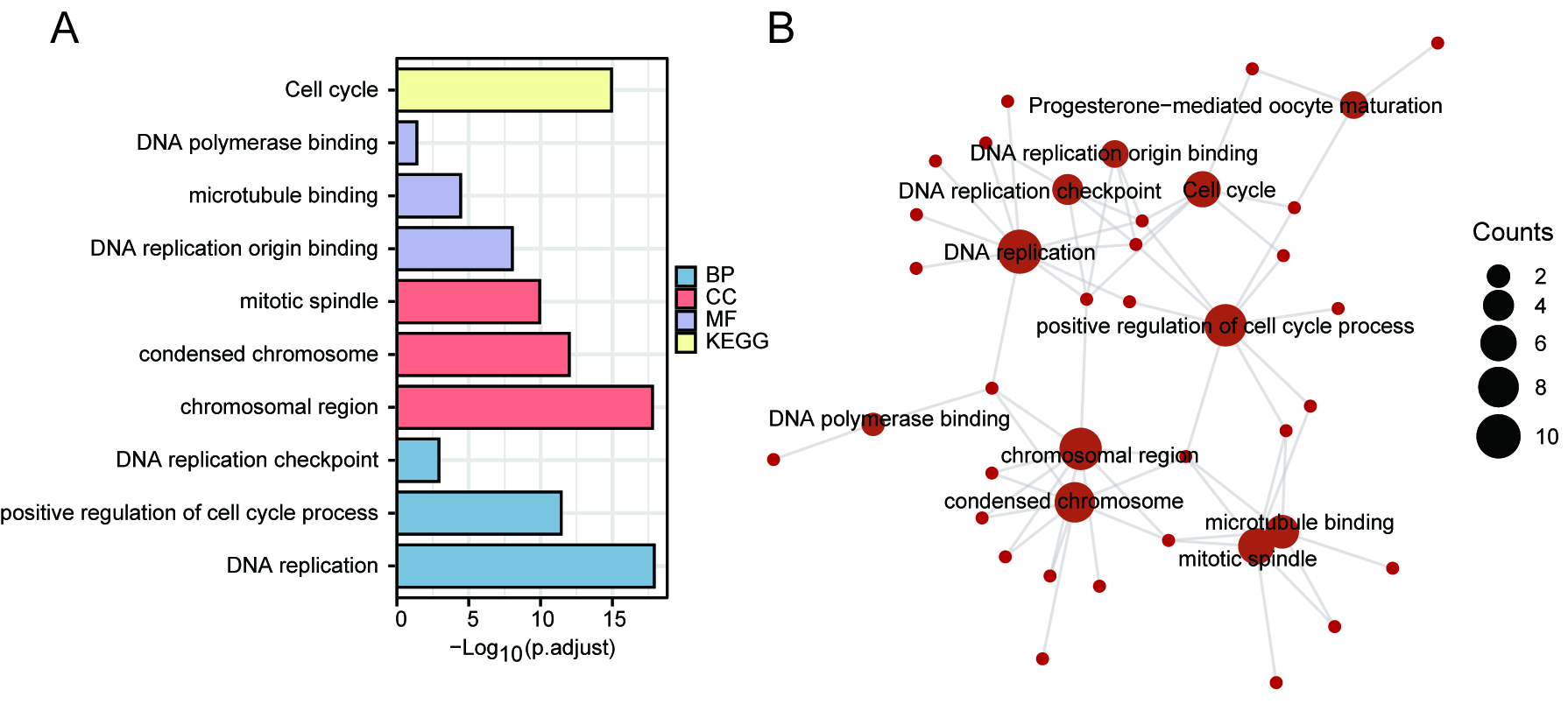

Supplement: Supplementary Figure 1 — Identification of CCP-related genes in cutaneous melanoma and their biological functions. (A). Histogram showing the significant biological process, cellular component, molecular function, and KEGG pathways enriched by CCP-related genes. (B). Networks showing the significant biological process, cellular component, molecular function, and KEGG pathways enriched by CCP-related genes. [file Image_1.tif]

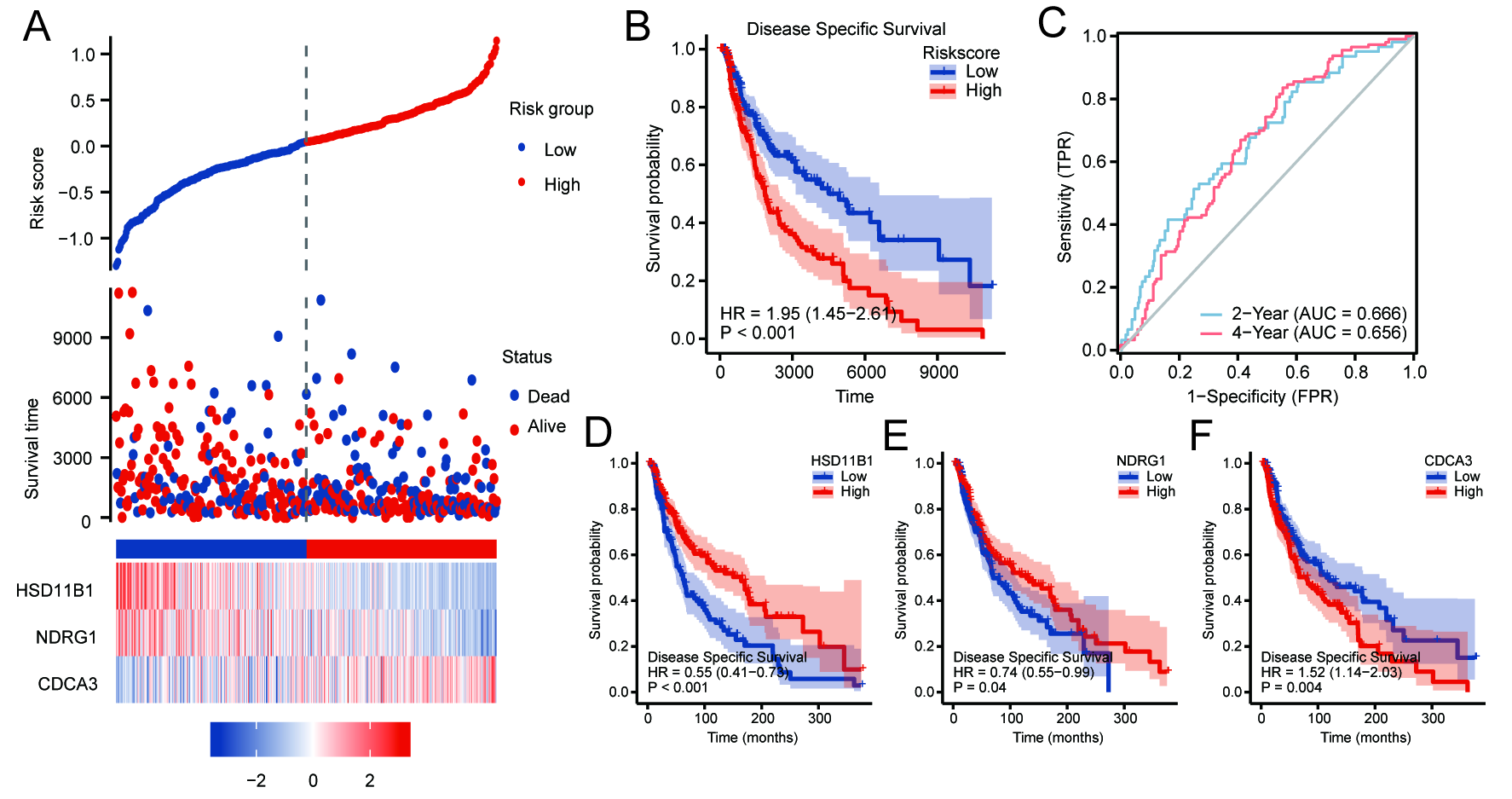

Supplement: Supplementary Figure 2 — Clinical relevance of CCP-related genes in patients with cutaneous melanoma in TCGA. To assess DSS outcome, (A) distribution of risk score, survival status and the expression of prognostic CCP-related genes; (B) Kaplan−Meier plot of the CCP-related gene signature and overall survival; and (C) ROC curves for 2- and 4-year survival prediction. Kaplan−Meier plot for the expression of (D) HSD11B1, (E) NDRG1 and (F) CDCA3. The hazard ratios (HRs) were evaluated using Cox proportional hazard models. OS: overall survival; ROC curve: receiver operating characteristic curve. [file Image_2.tif]

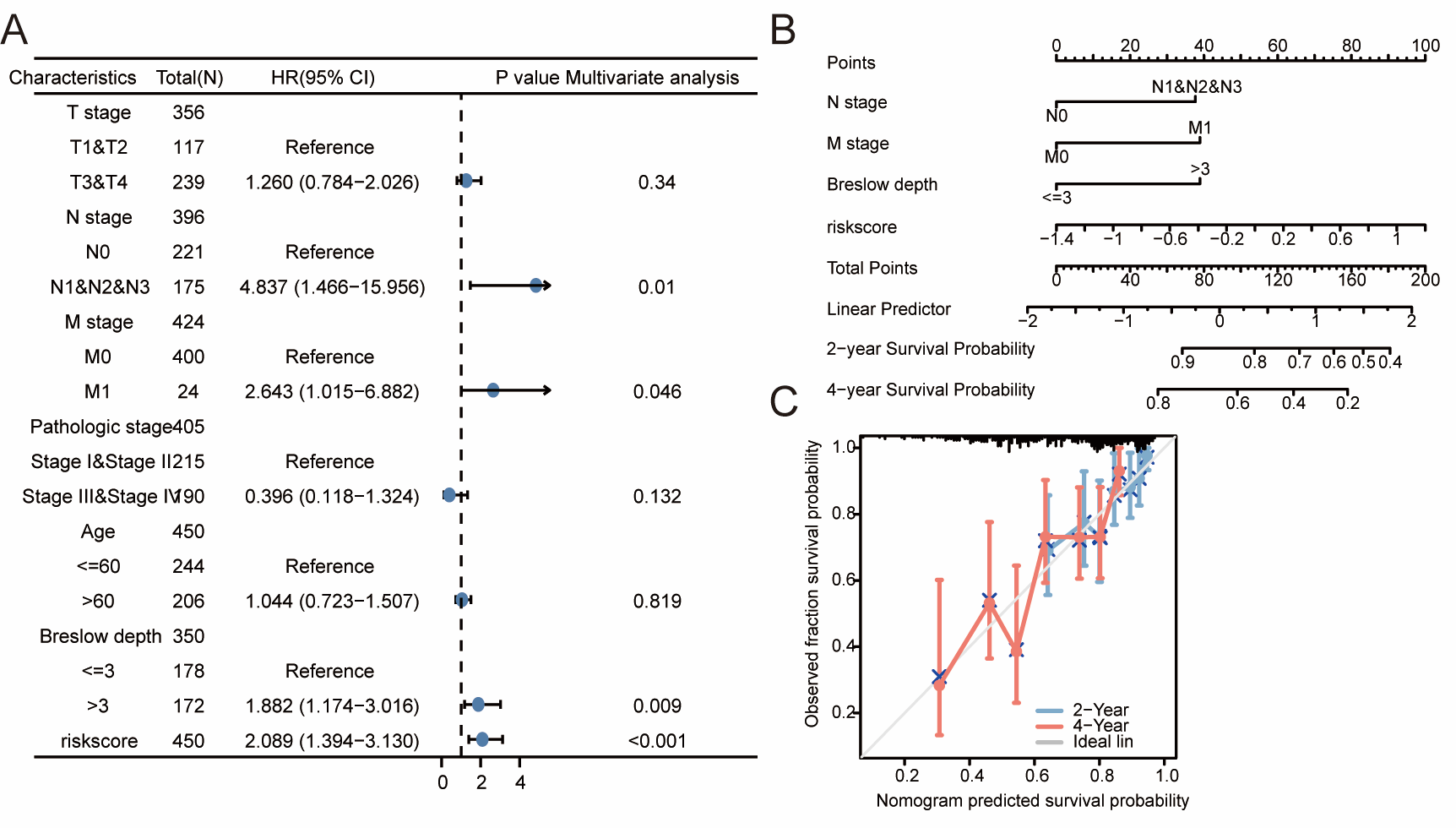

Supplement: Supplementary Figure 3 — The prognostic value of CCP-related genes (disease-specific survival) in diverse cutaneous melanoma (A). Forrest plot showing the results of multivariate Cox regression analysis. (B). Nomogram integrating the 3 CCP-related gene-based risk score, N stage, race, and Breslow depth. (C). Calibration plots of the nomogram for evaluating the probability of OS at 2 and 4 years. OS, overall survival. [file Image_3.tif]

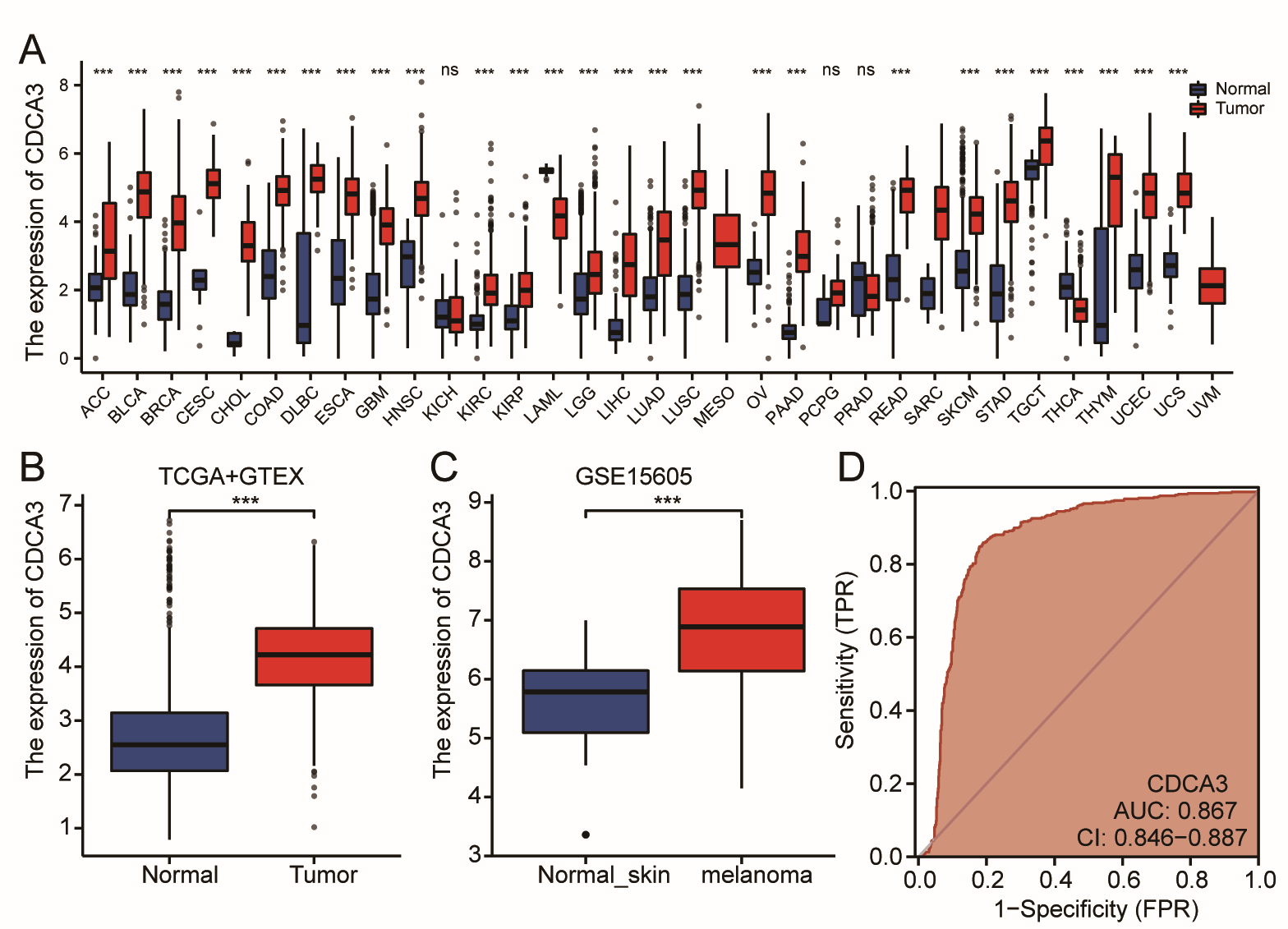

Supplement: Supplementary Figure 4 — The expression levels of CDCA3 in different cancer types and in cutaneous melanoma. (A) Increased or decreased CDCA3 expression in different cancer types compared with normal tissues in TCGA and GTEx databases. (B–D) The expression levels of CDCA3 in cutaneous melanoma. (E) ROC curve to investigate the value of CDCA3 in identifying cutaneous melanoma tissues. [file Image_4.tif]

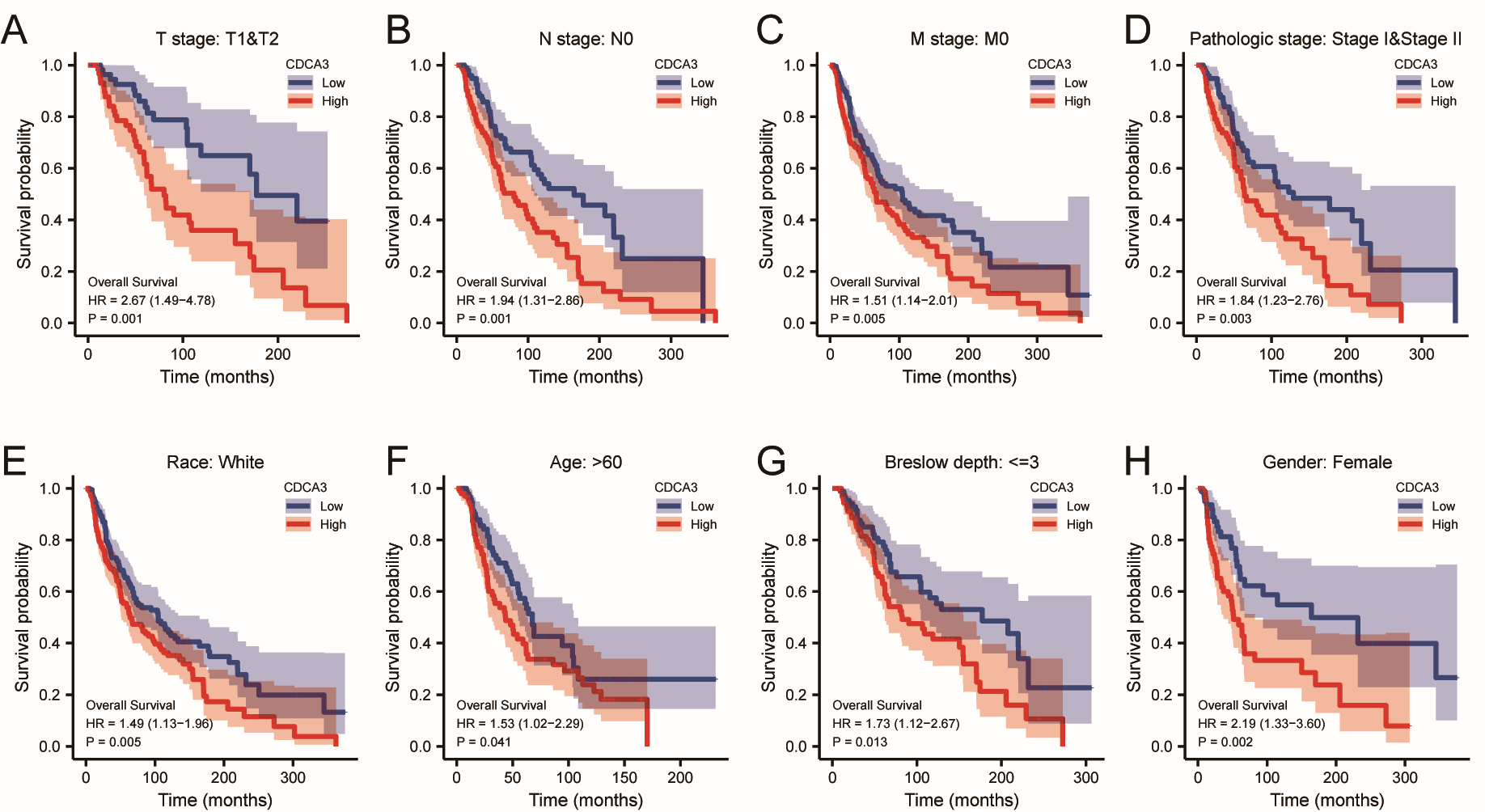

Supplement: Supplementary Figure 5 — Kaplan–Meier survival plots comparing the low and high expression of CDCA3 in cutaneous melanoma. (A–C) Survival curves of OS, DSS, and PFI between high and low CDCA3 expression in patients with cutaneous melanoma. (D–K) OS survival curves of T stages I–II, N0, M0, pathologic stage I−II, race of white, age > 60, female, Breslow depth< 3 subgroups between high and low CDCA3 expression in patients with cutaneous melanoma. OS, overall survival; DSS, disease-specific survival; PFI, progression-free interval. [file Image_5.tif]

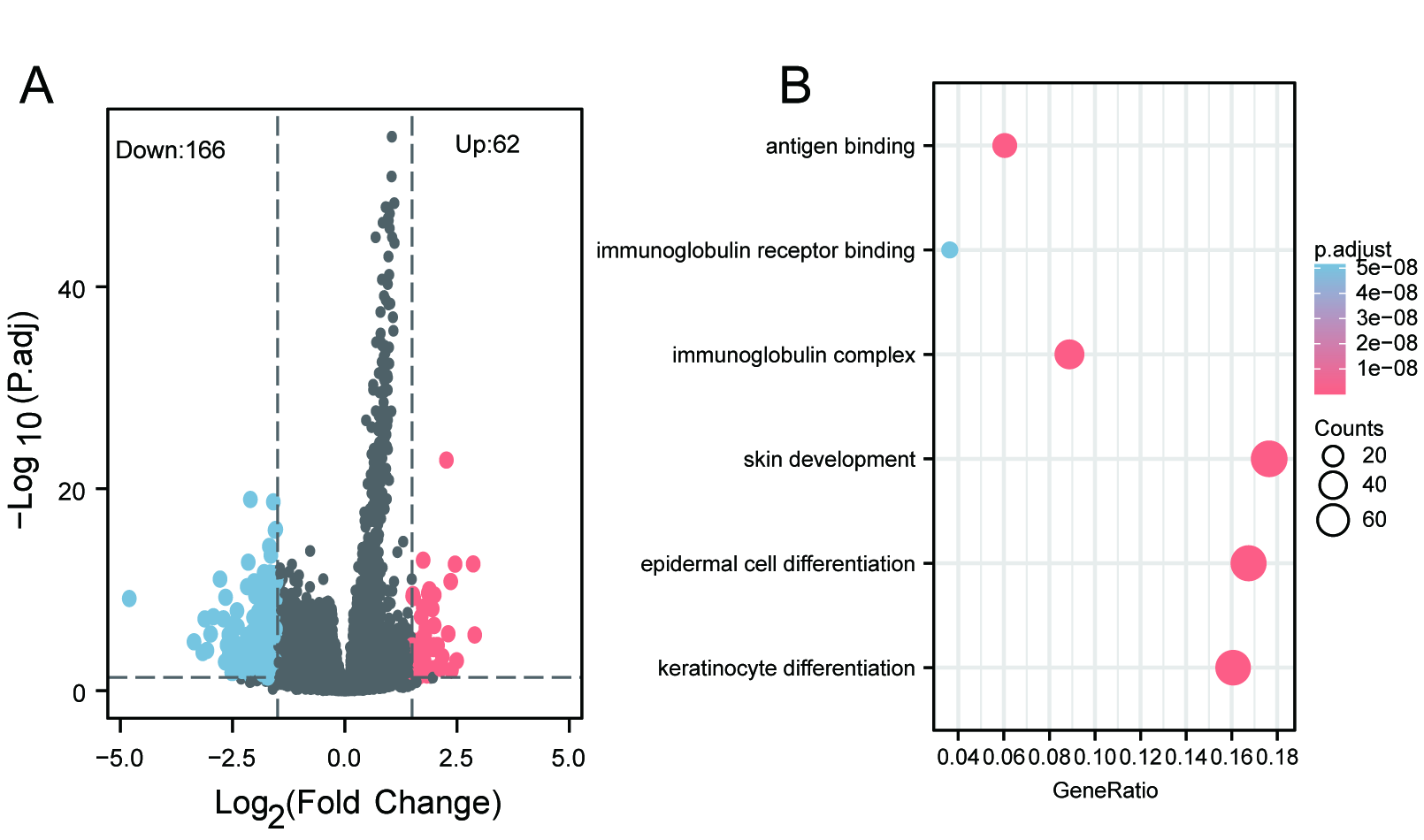

Supplement: Supplementary Figure 6 — Functional enrichment of CDCA3 in cutaneous melanoma. (A) Volcano plots of the DEGs between high and low CDCA3 expression in cutaneous melanoma. (B) Significantly enriched GO annotations of CDCA3-related genes in cutaneous melanoma. [file Image_6.tif]

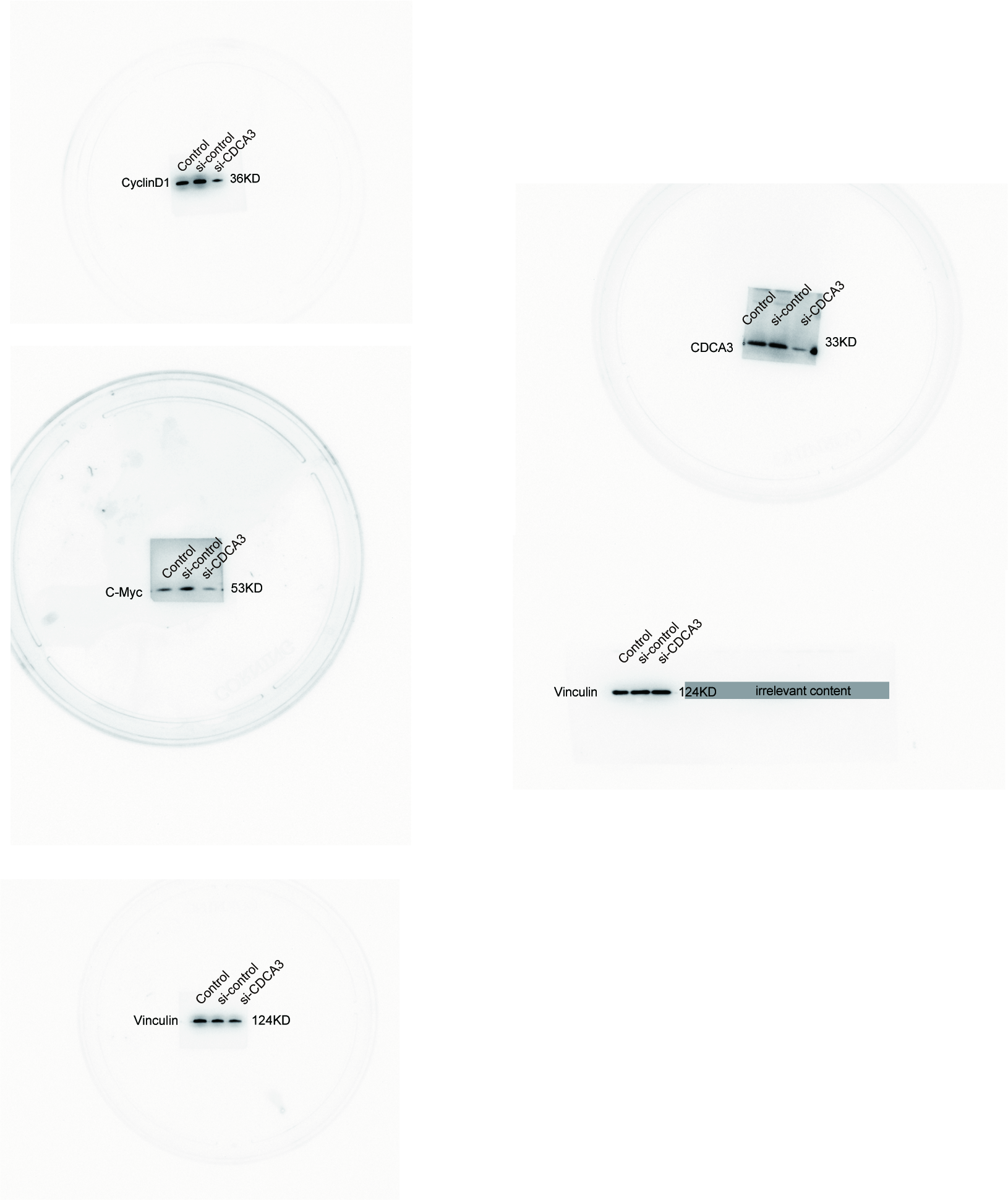

Supplement: Supplementary file 7 [file Image_7.tif]
