# Supplementary material for: CDCA3 is a prognostic biomarker for cutaneous melanoma and is connected with immune infiltration
Source: Front Oncol. 2023 Jan 11;12:1055308. doi: 10.3389/fonc.2022.1055308 (PMC9876620; doi:10.3389/fonc.2022.1055308)
Supplement: Supplementary file 8 [file Table_1.docx]

| The CCP gene sets of hallmarks of cancer | | | |  |
| --- | --- | --- | --- | --- |
| ABL1 | CDKN2B | POLD1 | KIF11 | TPD52L1 |
| ACVR1 | CDKN2C | POLE | KIF15 | TPX2 |
| ACVR1B | CDKN2D | PPP5C | KIF22 | TRIAP1 |
| AKAP8 | CDKN3 | PPP6C | KIF25 | TTK |
| ANAPC10 | CENPE | PRMT5 | KIF2C | TTN |
| ANAPC11 | CENPF | PRUNE2 | KNTC1 | UBE2C |
| ANAPC4 | CETN1 | PTPRC | KPNA2 | USH1C |
| ANAPC5 | CHEK1 | RAD1 | KRT7 | XRCC2 |
| ANLN | CHFR | RAD17 | LATS1 | ZNRD2 |
| APBB1 | CHMP1A | RAD21 | LATS2 | ZW10 |
| APBB2 | CIT | RAD50 | LIG3 | ZWINT |
| ATM | table | RAD51 | MAD2L1 | SNAI2 |
| AURKA | CLIP1 | RAD51B | MAD2L2 | SNTB1 |
| BCAT1 | CUL1 | RAD51D | MAP3K11 | SPARC |
| BIRC5 | CUL2 | RAD52 | MPHOSPH6 | SPOCK1 |
| BOLL | CUL3 | RAD54B | MPHOSPH9 | SPP1 |
| BRSK1 | CUL4A | RAD54L | MRE11 | TAGLN |
| BUB1 | CUL5 | RAN | MSH4 | TFPI2 |
| BUB1B | DBF4 | RB1 | MSH5 | TGFB1 |
| CCNA1 | DCTN2 | RCC1 | MYO16 | TGFBI |
| CCNA2 | DCTN3 | REC8 | NBN | TGFBR3 |
| CD28 | DDX11 | RINT1 | NCAPH | TGM2 |
| CDC16 | DLGAP5 | SKP2 | NDC80 | THBS1 |
| CDC23 | DMC1 | SMC1A | NEK2 | THBS2 |
| CDC25B | DUSP13 | SMC3 | NEK6 | THY1 |
| CDC25C | E2F1 | SMC4 | NOLC1 | TIMP1 |
| CDC27 | EGF | SPDYA | NPM2 | TIMP3 |
| CDC6 | EPGN | SPO11 | NUMA1 | TNC |
| CDC7 | EREG | STAG3 | NUSAP1 | TNFAIP3 |
| CDCA3 | ESPL1 | SUGT1 | P3H4 | TNFRSF11B |
| CDK10 | FOXN3 | SYCP1 | PAM | TNFRSF12A |
| CDK13 | FOXO4 | TAF1 | PBRM1 | TPM1 |
| CDK2 | GFI1 | TAF1L | PCBP4 | TPM2 |
| CDK2AP1 | GFI1B | TARDBP | PDS5B | TPM4 |
| CDK4 | GML | TBRG4 | PIM2 | VCAM1 |
| CDK6 | GSPT1 | TGFA | PIN1 | VCAN |
| CDKN1A | HSPA2 | TGFB1 | PKMYT1 | VEGFA |
| CDKN1B | INHBA | TIMELESS | PLK1 | VEGFC |
| CDKN1C | KATNA1 | TIPIN | PML | VIM |
| CDKN2A | KHDRBS1 | TOP3A | POLA1 |  |
| NDRG1 | WNT5A | HSD11B1 | WIPF1 |  |
