# Supplementary material for: CDCA3 is a prognostic biomarker for cutaneous melanoma and is connected with immune infiltration
Source: Front Oncol. 2023 Jan 11;12:1055308. doi: 10.3389/fonc.2022.1055308 (PMC9876620; doi:10.3389/fonc.2022.1055308)
Supplement: Supplementary file 9 [file Table_2.docx]

| Gene Symbol | Gene ID | Median (Tumor) | Median (Normal) | Log2(Fold Change) | Adj.P |
| --- | --- | --- | --- | --- | --- |
| PYCR1 | ENSG00000183010.16 | 57.348 | 4.415 | 3.43 | 1.1E-272 |
| DTL | ENSG00000143476.17 | 11.68 | 0.82 | 2.801 | 1.3E-232 |
| CDC6 | ENSG00000094804.9 | 11.22 | 0.785 | 2.775 | 5E-239 |
| KPNA2 | ENSG00000182481.8 | 85.942 | 11.995 | 2.742 | 3.5E-256 |
| EXO1 | ENSG00000174371.16 | 7.51 | 0.53 | 2.476 | 9.9E-246 |
| PAFAH1B3 | ENSG00000079462.7 | 62.318 | 10.765 | 2.428 | 2.3E-249 |
| CDC45 | ENSG00000093009.9 | 12.25 | 1.5 | 2.406 | 1.9E-211 |
| TRIP13 | ENSG00000071539.13 | 9.5 | 1.12 | 2.308 | 9.4E-217 |
| TUBA1B | ENSG00000123416.15 | 1082.836 | 221.152 | 2.287 | 3.6E-247 |
| UHRF1 | ENSG00000276043.4 | 9.73 | 1.32 | 2.209 | 5E-183 |
| PKMYT1 | ENSG00000127564.16 | 23.821 | 4.47 | 2.182 | 5.5E-167 |
| MND1 | ENSG00000121211.7 | 6.85 | 0.745 | 2.169 | 1.3E-229 |
| SPC24 | ENSG00000161888.11 | 8.52 | 1.33 | 2.031 | 2.7E-213 |
| TIMELESS | ENSG00000111602.11 | 14.29 | 4.49 | 1.944 | 2.1E-150 |
| KIF18B | ENSG00000186185.13 | 5.49 | 1.33 | 1.944 | 7.6E-122 |
| ZBTB12 | ENSG00000204366.3 | 5.01 | 1.2 | 1.916 | 5.8E-151 |
| GINS2 | ENSG00000131153.8 | 7.48 | 1.32 | 1.87 | 6.9E-197 |
| CDC25A | ENSG00000164045.11 | 4.02 | 0.39 | 1.853 | 1.1E-210 |
| SKA1 | ENSG00000154839.9 | 3.9 | 0.36 | 1.849 | 3.6E-214 |
| INCENP | ENSG00000149503.12 | 23.65 | 8.52 | 1.839 | 2.8E-127 |
| RAD51 | ENSG00000051180.16 | 9.08 | 1.85 | 1.823 | 2.4E-168 |
| CHAF1B | ENSG00000159259.7 | 5.86 | 1.74 | 1.79 | 4.7E-146 |
| PAQR4 | ENSG00000162073.13 | 29.84 | 11.485 | 1.771 | 1.9E-104 |
| CDK4 | ENSG00000135446.16 | 181.483 | 53.891 | 1.733 | 8E-203 |
| ORC1 | ENSG00000085840.12 | 3.49 | 0.88 | 1.722 | 8.1E-123 |
| CDCA3 | ENSG00000111665.11 | 17.67 | 4.675 | 1.718 | 3.6E-147 |
| SPAG5 | ENSG00000076382.16 | 18.81 | 7.4 | 1.704 | 2.7E-104 |
| RACGAP1 | ENSG00000161800.12 | 20.11 | 8.04 | 1.689 | 7.49E-94 |
| WDHD1 | ENSG00000198554.11 | 6.04 | 2.05 | 1.673 | 6.38E-98 |
| NCAPG2 | ENSG00000146918.19 | 14.02 | 3.755 | 1.659 | 1.5E-141 |
| NCAPD2 | ENSG00000010292.12 | 29.539 | 12.495 | 1.644 | 1.2E-111 |
| CENPO | ENSG00000138092.10 | 8.44 | 3.245 | 1.619 | 2E-135 |
| CENPI | ENSG00000102384.13 | 3.26 | 0.41 | 1.595 | 1.1E-182 |
| CIT | ENSG00000122966.13 | 8.46 | 3.36 | 1.583 | 1.58E-67 |
| FANCI | ENSG00000140525.17 | 17.8 | 5.425 | 1.549 | 3.1E-128 |
| CKAP2L | ENSG00000169607.12 | 4.03 | 0.72 | 1.548 | 9.1E-127 |
| LMNB2 | ENSG00000176619.10 | 28.989 | 9.525 | 1.511 | 2E-165 |
| ECT2 | ENSG00000114346.13 | 8.85 | 3.795 | 1.505 | 1.6E-51 |
| NRM | ENSG00000137404.14 | 33.52 | 15.845 | 1.501 | 1.75E-95 |
| STK26 | ENSG00000134602.15 | 1.4 | 4.67 | -1.524 | 1.05E-50 |
| FMO3 | ENSG00000007933.12 | 0.6 | 2.39 | -1.583 | 5.89E-44 |
| C7 | ENSG00000112936.18 | 2.57 | 12.335 | -1.901 | 1.46E-27 |
| PLBD1 | ENSG00000121316.10 | 5.18 | 23.325 | -1.977 | 6.48E-73 |
| NDRG1 | ENSG00000104419.14 | 100.349 | 421.59 | -2.06 | 4.23E-68 |
| CHI3L1 | ENSG00000133048.12 | 10.12 | 76.914 | -2.809 | 2.12E-73 |
| C15orf48 | ENSG00000166920.10 | 4.99 | 41.254 | -2.818 | 1.4E-106 |
| AOX1 | ENSG00000138356.13 | 0.81 | 17.425 | -3.348 | 4.6E-202 |
| HSD11B1 | ENSG00000117594.9 | 1.08 | 21.995 | -3.467 | 9.2E-219 |
| MAPK13 | ENSG00000156711.16 | 3.29 | 59.249 | -3.812 | 2.1E-284 |
| MT1X | ENSG00000187193.8 | 65.018 | 1340.494 | -4.345 | 4.4E-231 |
| SPINT2 | ENSG00000167642.12 | 7.91 | 265.045 | -4.9 | 2.6E-304 |
